# PRRX1 induced by BMP signaling decreases tumorigenesis by epigenetically regulating glioma‐initiating cell properties via DNA methyltransferase 3A

**DOI:** 10.1002/1878-0261.13051

**Published:** 2021-07-16

**Authors:** Ryo Tanabe, Kohei Miyazono, Tomoki Todo, Nobuhito Saito, Caname Iwata, Akiyoshi Komuro, Satoshi Sakai, Erna Raja, Daizo Koinuma, Masato Morikawa, Bengt Westermark, Carl‐Henrik Heldin

**Affiliations:** ^1^ Department of Molecular Pathology Graduate School of Medicine The University of Tokyo Japan; ^2^ Department of Medical Biochemistry and Microbiology Science for Life Laboratory Uppsala University Sweden; ^3^ Division of Innovative Cancer Therapy The Institute of Medical Science The University of Tokyo Japan; ^4^ Department of Neurosurgery Graduate School of Medicine The University of Tokyo Japan; ^5^ Department of Immunology, Genetics and Pathology Science for Life Laboratory Rudbeck Laboratory Uppsala University Sweden; ^6^ Present address: Department of Biochemistry Faculty of Medicine Kindai University Osaka Japan; ^7^ Present address: Laboratory for Safety Assessment & ADME Pharmaceuticals Research Center Asahi Kasei Pharma Corporation Shizuoka Japan

**Keywords:** BMP, cancer‐initiating cell, CD133, DNA methyltransferase, glioblastoma, PRRX1

## Abstract

Glioma‐initiating cells (GICs), a major source of glioblastoma recurrence, are characterized by the expression of neural stem cell markers and the ability to grow by forming nonadherent spheres under serum‐free conditions. Bone morphogenetic proteins (BMPs), members of the transforming growth factor‐β family, induce differentiation of GICs and suppress their tumorigenicity. However, the mechanisms underlying the BMP‐induced loss of GIC stemness have not been fully elucidated. Here, we show that paired related homeobox 1 (PRRX1) induced by BMPs decreases the CD133‐positive GIC population and inhibits tumorigenic activity of GICs *in vivo*. Of the two splice isoforms of PRRX1, the longer isoform, pmx‐1b, but not the shorter isoform, pmx‐1a, induces GIC differentiation. Upon BMP stimulation, pmx‐1b interacts with the DNA methyltransferase DNMT3A and induces promoter methylation of the *PROM1* gene encoding CD133. Silencing *DNMT3A* maintains *PROM1* expression and increases the CD133‐positive GIC population. Thus, pmx‐1b promotes loss of stem cell‐like properties of GICs through region‐specific epigenetic regulation of CD133 expression by recruiting DNMT3A, which is associated with decreased tumorigenicity of GICs.

## Graphical abstract

BMPs induce differentiation and apoptosis of glioma‐initiating cells (GICs). Here, we identified PRRX1 as a target of BMPs in GICs. Of the two splice isoforms, only pmx‐1b induced GIC differentiation. Through interaction with DNMT3A, pmx‐1b induced the methylation of the *PROM1* gene promoter and suppressed the CD133^+^ GIC population. Thus, site‐specific epigenetic regulation plays a critical role in the differentiation of GICs.

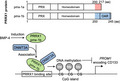

**Abbreviations**
ALK: activin receptor‐like kinase
bFGF: basic fibroblast growth factor
BMP: bone morphogenetic protein
BMPR: BMP receptor
ChIP: chromatin immunoprecipitation
DNMT: DNA methyltransferase
EGF: epidermal growth factor
EGFP: enhanced green fluorescent protein
GIC: glioma‐initiating cell
HGCC: Human Glioblastoma Cell Culture
hOPC: human oligodendrocyte progenitor cell
NSC: neural stem cell
PCR: polymerase chain reaction
PI3K: phosphoinositide 3’-kinase
pmx1: paired mesoderm homeobox 1
PRRX1: paired related homeobox 1
qRT-PCR: quantitative real-time PCR
shRNA: short hairpin RNA
TGF-β: transforming growth factor‐β

## 1 Introduction

Glioblastoma is the most malignant form of adult brain tumors. Despite recent advances and new treatment strategies, the median survival of patients with glioblastoma remains <20 months [1, 2, 3]. Recent efforts to target abnormalities in the signaling pathways in glioblastoma cells have not been successful in improving treatment efficacy [4, 5].

Glioma‐initiating cells (GICs) play central roles in the initiation, progression, recurrence, tumor heterogeneity, and drug and radiation resistance of glioblastoma [6, 7, 8]. GICs are cellular subpopulations of glioblastoma that express neural stem and progenitor cell markers, such as *PROM1* (encoding CD133), *OLIG2*, and *SOX2* [9, 10]. CD133 is a cell surface glycoprotein expressed on neural stem cells (NSCs), and its expression decreases as cells differentiate. CD133 is thus a stem cell marker that is frequently used to isolate GICs [6, 7]. Moreover, loss of function of CD133 has been shown to impair self‐renewal and tumorigenic capacity of GICs [11], suggesting critical roles of CD133 in the pathogenesis of glioblastoma.

Members of the transforming growth factor‐β (TGF‐β) family are multifunctional proteins, involved in pathogenesis of various diseases, including cancer [12, 13]. Bone morphogenetic proteins (BMPs), members of the TGF‐β family, regulate the growth and differentiation of a wide variety of cells, including different types of cancer cells [14]. BMPs activate BMP receptor–SMAD signaling pathways and non‐SMAD pathways [15]. BMP signaling regulates normal embryonic neural development through the activation of BMP type I receptors, including activin receptor‐like kinase (ALK)‐2, ALK‐3/BMP receptor type IA (BMPRIA), and ALK‐6/BMPRIB. BMPs inhibit the tumorigenic potential of human GICs by inducing cell differentiation, cell cycle arrest, and apoptosis [16, 17, 18, 19, 20] and render GICs more susceptible to conventional chemotherapies [21]. Expression of *BMP4* is decreased in high‐grade glioma compared to low‐grade glioma, and patients with high *BMP4* expression show significantly better prognosis than those with low *BMP4* expression [22]. Thus, BMP signaling components are considered important biomarkers and potential therapeutic targets for glioblastoma.

Antitumorigenic activities of BMPs on GICs have been reported by many investigators [21, 23]; however, some GICs respond to BMP treatment, whereas others escape the differentiation‐inducing effect of BMP and fail to undergo terminal cell cycle arrest despite active BMP signaling [24, 25]. A fraction of genes that are silenced during the course of BMP‐induced astrocyte differentiation of NSCs fail to be downregulated in the latter population of GICs [24]. Thus, considering the heterogeneity of glioblastoma cells, the mechanisms by which BMPs affect GICs should be further investigated.

Various molecules have been reported to function as downstream components in the BMP signaling pathways in glioblastoma. Transcription factors such as Snail, p21^WAF1/CIP1^, and Distal‐less homeobox 2 have been shown to be induced by BMP signaling and play tumor suppressive roles in glioblastoma [19, 20, 26, 27]. In addition, a decrease in cyclin D1 is involved in growth inhibition and the induction of Bax and inhibition of Bcl‐2 and Bcl‐xL in apoptosis [18]. Moreover, certain extracellular or cell surface molecules regulate the action of BMPs in glioblastoma. Gremlin1, an extracellular antagonist of BMPs, is secreted by GICs and maintains their stem cell‐like properties by inhibiting endogenous BMP signaling [19]. We showed that, among the three BMP type I receptors expressed in GICs, ALK‐2 plays a major role in apoptosis regulation [20] and that cooperation with EphA6 tyrosine kinase receptor sensitizes some resistant GICs for BMP‐induced apoptosis [28]. However, because of the heterogeneity of glioblastoma cells, certain other BMP target molecules may also be critically involved in differentiation and apoptosis of GICs, and it remains to be elucidated how the stem cell‐like properties of GICs are regulated by BMPs.

By analyzing RNA‐sequencing (RNA‐seq) data [20], we have identified certain genes specifically induced by BMPs in GICs. We show that paired related homeobox 1 (PRRX1; also known as pmx1 (paired mesoderm homeobox 1)) is induced by BMP‐4 stimulation and promotes differentiation of GICs by decreasing the CD133‐positive cell population. Intriguingly, the DNA methyltransferase 3A (DNMT3A) interacts with one of the splice isoforms of PRRX1 and induces methylation of the *PROM1* gene promoter, leading to a decrease in the CD133‐positive GIC population. Thus, our findings suggest a critical role of PRRX1 induction by BMP‐4 in regulation of the stem cell‐like properties of GICs through epigenetic regulation of the *PROM1* gene by DNA methyltransferase.

## 2 Materials and methods

### 2.1 Cell culture and reagents

TGS‐01 and TGS‐04 cells were obtained during surgery from consenting patients with glioblastoma multiforme (grade IV), as approved by the Institutional Review Board at the University of Tokyo Hospital (characteristics of the cells are listed in Table S1), as described previously [20, 29], and were cultured in Dulbecco’s Eagle’s medium/F12 containing B27 supplement (Thermo Fisher Scientific, Waltham, MA, USA) and 20 ng·mL^−1^ of epidermal growth factor (EGF, PeproTech, Cranbury, NJ, USA) and basic fibroblast growth factor (bFGF, PeproTech). The use of patient‐derived glioma cells (TGS‐01 and TGS‐04) was approved by the Research Ethics Committee of the Graduate School of Medicine, The University of Tokyo. Patient‐derived glioblastoma cells (U3005MG and U3024MG), obtained in accordance with protocols approved by the regional ethical review board, were acquired from the Human Glioblastoma Cell Culture (HGCC) resource (https://www.hgcc.se) at the Department of Immunology, Genetics and Pathology, Uppsala University, Uppsala, Sweden [30]. U3005MG and U3024MG were cultured in Dulbecco’s Eagle’s medium/F12 and Neurobasal medium (Thermo Fisher Scientific), enriched with B‐27 supplement, N‐2 supplement (Thermo Fisher Scientific), and 20 ng·mL^−1^ of EGF and bFGF. Recombinant human BMP‐4 was purchased from R&D Systems (Minneapolis, MN, USA) and used at 30 ng·mL^−1^. The antibodies used were as follows: anti‐PRRX1 (ab67631, Abcam, Cambridge, UK), anti‐PRRX1 (NBP1‐06067, Novus Biological, Centennial, CO, USA), anti‐PRRX1 C‐terminal (ab174201, Abcam), anti‐α‐tubulin (T6199, Sigma‐Aldrich, Merck, Burlington, MA, USA), anti‐FLAG M2 (F3165, Sigma‐Aldrich, Merck), anti‐CD133/2 (293C2, Miltenyi Biotec, Auburn, CA, USA), IgG2b (IS6‐11E5.11, Miltenyi Biotec), anti‐DNMT3A (ab2850 for immunoblotting and ab13888 for immunoprecipitation, Abcam), anti‐SMAD1 (9743, Cell Signaling Technology, Danvers, MA, USA), anti‐phospho‐SMAD1/5 (9516, Cell Signaling Technology), mouse IgG1 isotype control (MAB002, R&D Systems), and normal rabbit IgG (sc‐2027, Santa Cruz, Dallas, TX, USA).

### 2.2 Quantitative real‐time polymerase chain reaction (qRT‐PCR) analysis

Total RNA was extracted from GICs using RNeasy Mini Kit (Qiagen, Hilden, Germany). Complementary DNA was synthesized using a PrimeScript II First Strand cDNA Synthesis Kit (Takara Bio, Shiga, Japan) or an iScript cDNA Synthesis Kit (Bio‐Rad, Hercules, CA, USA). Gene expression levels were quantified on Step One Plus Real‐Time PCR Systems (Thermo Fisher Scientific) or CFX Connect Real‐Time PCR Detection System (Bio‐Rad) with FastStart Universal SYBR Green Master Mix (Roche Applied Science, Basel, Switzerland) or KAPA SYBR FAST qPCR Kit Master Mix (Kapa Biosystems, London, UK). Primer sets are listed in Table S2.

### 2.3 Plasmid construction

Enhanced green fluorescent protein (EGFP), FLAG‐tagged PRRX1 pmx‐1a and pmx‐1b, and *firefly* luciferase (Promega, Madison, WI, USA) cDNAs were cloned into pENTR201 vector (Thermo Fisher Scientific), and recombination between pENTR201 and CSII‐EF‐RfA vectors was catalyzed by LR clonase (Thermo Fisher Scientific). DNA sequences encoding short hairpin RNAs (shRNAs) were inserted into pENTR4‐H1 vector, and LR reaction between pENTR4‐H1 and CS‐RfA‐CMV‐PuroR vectors was performed. The sequences targeted by shRNAs are listed in Table S3. *PROM1* promoter P1 (−1900 to −1) was cloned as previously described [31]. The *PROM1* promoter P1 and *firefly* luciferase (Fluc) genes were subcloned between *SalII* and *XbaI* sites of the pENTR4‐H1 vector to generate pENTR4‐PROM1‐Fluc. The *EGFP* gene on CS‐RfA‐CG was replaced by the *renilla* luciferase gene to generate CS‐RfA‐CMV‐Rluc, and LR reaction between the pENTR4‐PROM1‐Fluc and CS‐RfA‐CMV‐Rluc vectors was performed. *PROM1* promoter P1 (−1010 to −1) was subcloned as a deletion mutant. Mutagenesis of the putative PRRX1‐binding sites on *PROM1* promoter P1 was introduced by site‐specific PCR with the oligonucleotides shown in Table S4.

### 2.4 Lentiviral transduction

The indicated vector plasmids, pCAG‐HIVgp and pCMV‐VSV‐G‐Rev, were transduced into HEK293FT cells using Lipofectamine 2000 (Thermo Fisher Scientific). Lentiviral particles were concentrated with Lenti‐X Concentrator (Takara Bio). Lentiviral vectors, pCSII‐EF‐RfA, pENTR4‐H1, pCAG‐HIVgp, and pCMV‐VSV‐G‐Rev, were gifts from late H. Miyoshi (formerly RIKEN, Saitama, Japan).

### 2.5 Luciferase reporter assay

Cells stably expressing *firefly* and *renilla* luciferase were lysed, and luciferase activities were determined using a Dual‐luciferase Reporter Assay System (Promega) on Mithras LB940 (Berthold Technologies, Bad Wildbad, Germany). The values of *firefly* luciferase activity were normalized to those of *renilla* luciferase activity.

### 2.6 Sphere formation assay

TGS‐01 or TGS‐04 cells were seeded on ultra‐low attachment microplates (Corning, Kennebunk, ME, USA) at a density of 1–50 cells per well by using Cell Sorter SH800 Series (Sony Corporation, Tokyo, Japan) and then cultured for 7 days. Cell masses with more than 50 µm in diameter were considered as spheres.

### 2.7 Immunoblot analysis

Cells were lysed with a buffer containing 50 mm Tris/HCl (pH 8.0), 150 mm NaCl, 0.1% SDS, 0.5% sodium deoxycholate, and 1% Triton X‐100. Cleared cell lysates were subjected to SDS/PAGE and transferred onto a Fluoro Trans W Membrane (Pall, East Hills, NY, USA). Immunoblotting was performed using the indicated antibodies, and chemiluminescence was detected using ImageQuant LAS4000 (Fujifilm, Tokyo, Japan).

### 2.8 Immunoprecipitation followed by immunoblotting

For immunoprecipitation, cells were lysed with a lysis buffer (20 mm Tris/HCl (pH 7.4), 10 mm KCl, 10 mm MgCl_2_, 2 mm EDTA, 10% glycerol, 1% Triton X‐100, 2.5 mm β‐glycerophosphate, 1 mm NaF, 1 mm dithiothreitol, and cOmplete Protease Inhibitor Cocktail (Sigma‐Aldrich, Merck)), followed by resolubilization by the addition of NaCl at a final concentration of 420 mm [32]. Antibodies bound to Dynabeads M‐280 sheep anti‐mouse IgG or Dynabeads Protein A (Thermo Fisher Scientific) were mixed with cell lysates at 4 °C. Dynabeads were washed with RIPA buffer containing 50 mm Tris/HCl (pH 8.0), 150 mm NaCl, 0.1% SDS, 0.5% sodium deoxycholate, and 1% Nonidet P‐40 and boiled at 95 °C for 5 min. Samples were then subjected to SDS/PAGE, followed by immunoblot analysis.

### 2.9 Chromatin immunoprecipitation (ChIP)

Cells were fixed with 1% formaldehyde for 10 min, whereafter glycine was added to a final concentration of 0.125 m to stop the reaction. After washing with ice‐cold phosphate‐buffered saline, the cells were scraped, pelleted, and resuspended in sonication/elution buffer (50 mm Tris/HCl (pH 8.1), 1% SDS, and 10 mm EDTA, containing cOmplete Protease Inhibitor Cocktail). After sonication, a solution containing the complex of anti‐FLAG antibody and Dynabeads M‐280 sheep anti‐mouse IgG, mixed with immunoprecipitation buffer (20 mm Tris/HCl (pH 8.0), 150 mm NaCl, 2 mm EDTA, 1% Triton X‐100, and cOmplete Protease Inhibitor Cocktail), was added, followed by centrifugation at 4 °C. The beads were then washed with washing buffer (50 mm HEPES‐KOH (pH 7.0), 500 mm LiCl, 1 mm EDTA, 0.7% sodium deoxycholate, and 1% Nonidet P‐40) and TE buffer (10 mm Tris/HCl (pH 8.0), 1 mm EDTA (pH 8.0)), and DNA was eluted in the sonication/elution buffer at 65 °C for 16 h. Genomic DNA was then extracted with a PCR purification kit (Qiagen), and DNA fragments were analyzed using the primers shown in Table S5, FastStart Universal SYBR Green Master Mix (Roche Applied Science, Penzberg, Germany), and Step One Plus Real‐Time PCR Systems.

### 2.10 Flow cytometric analysis

Cells were dissociated and labeled with a phycoerythrin‐conjugated anti‐CD133/2 (293C2) antibody (Miltenyi Biotec). The expression of CD133 on cell surfaces was analyzed on BD LSR II Flow Cytometer (BD Biosciences, Franklin Lakes, NJ, USA) or Cell Sorter SH800 Series (Sony Corporation) with FlowJo (BD Biosciences).

### 2.11 Mouse tumor model

All animal experiments were approved by and carried out according to the guidelines of the Animal Ethics Committee of the Graduate School of Medicine, The University of Tokyo. A total of 5 × 10^4^ viable cells were injected over bregma 2 mm to the right of the sagittal suture and 3 mm below the surface of the skulls of 5‐week‐old female BALB/c nu/nu mice (Sankyo Lab Service, Tokyo, Japan). For *in vivo* bioluminescence imaging, D‐luciferin (Promega) was injected intraperitoneally and photons were detected using NightOwl LB983 (Berthold Technologies). The mice were euthanized when a weight loss > 20% or neurological signs such as hemiparesis were observed.

### 2.12 DNA methylation analysis

Genomic DNA was isolated from TGS‐01 and TGS‐04 cells, and bisulfite treatment was performed using an EpiTect Bisulfite Conversion Kit (Qiagen). Using the bisulfite‐treated genomic DNA as a template, PCR was carried out using EpiTaq HS (Takara Bio). The PCR products were analyzed by sequencing or agarose gel electrophoresis. The primers used for the PCR are listed in Tables S6 and S7.

### 2.13 Statistical analysis

Student’s *t*‐test, Tukey’s test, and the log‐rank test were performed using statistical software r (http://www.R‐project.org).

## 3 Results

### 3.1 PRRX1 is induced by BMP‐4 and directly regulates the *PROM1* promoter in GICs

**Fig. 1:**
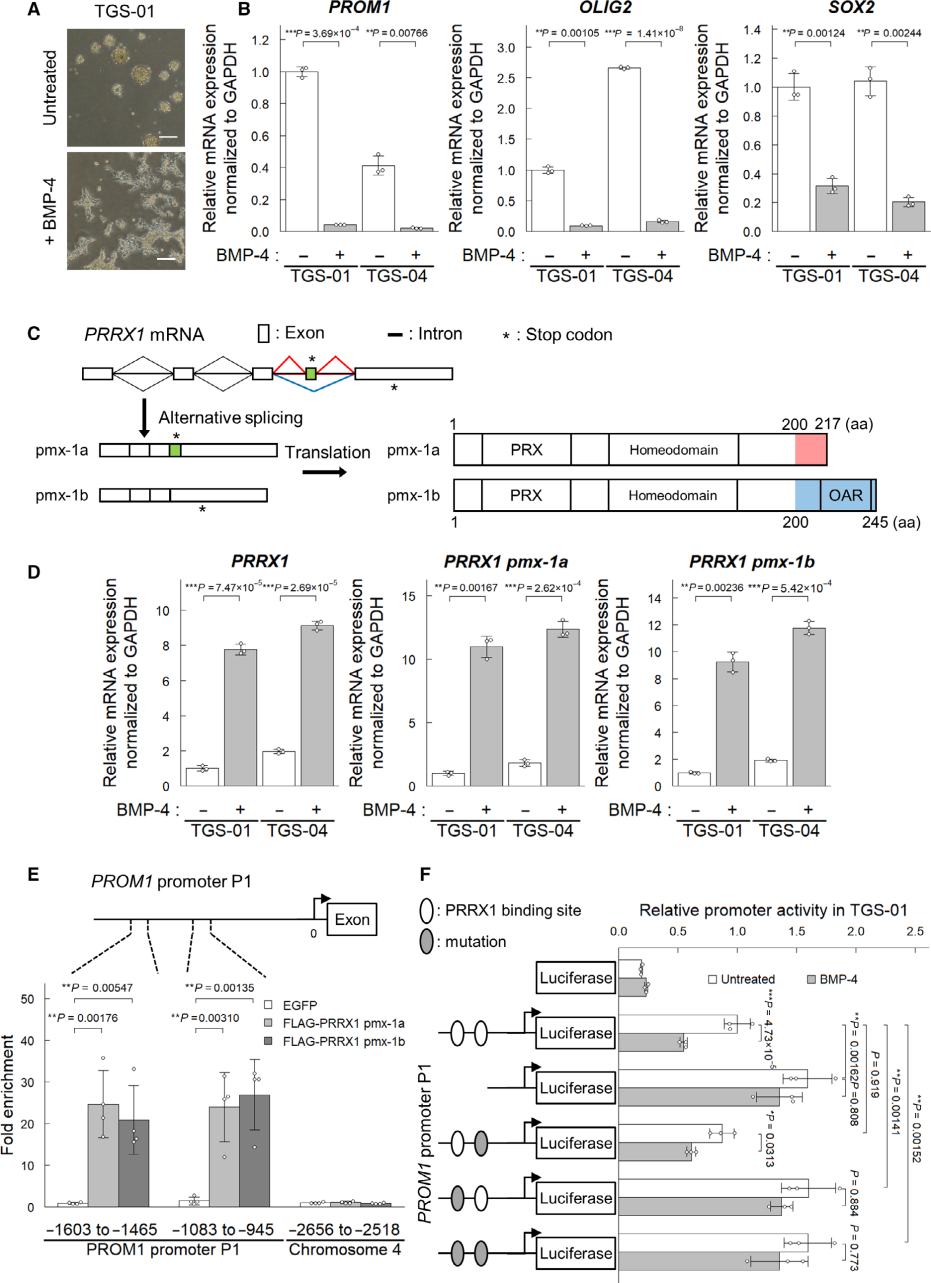
BMP‐4 induces differentiation of GIC and upregulates the expression of *PRRX1*. TGS‐01 and TGS‐04 cells were treated with or without BMP‐4 (30 ng·mL^−1^) for 72 h, except for panel (A). (A) Failure of sphere formation of TGS‐01 cells in the presence of BMP‐4 for 5 days. Scale bars: 200 µm. (B) Downregulation of glioma stem cell markers (*n* = 3 biological replicates). (C) A scheme of the splice isoforms of PRRX1. (D) Induction of the expression of two splice isoforms of PRRX1 in TGS‐01 and TGS‐04 cells by BMP‐4 stimulation (*n* = 3 biological replicates). (E) ChIP‐qPCR analysis of the binding of pmx‐1a and pmx‐1b on the *PROM1* promoter in TGS‐01 cells stably expressing FLAG‐tagged PRRX1 proteins (*n* = 4 biological replicates). (F) Relative promoter activity measured in TGS‐01 cells treated with or without BMP‐4 by dual‐luciferase assay (*n* = 3 biological replicates). The *PROM1* promoter was subcloned, and the AT‐rich sequence of PRRX1‐binding sites was deleted or converted into a GC‐rich sequence. The graphs in panels (B) and (D–F) represent mean ± SD of biological replicates. The *P*‐values were determined by Student’s *t*‐test (B) or Tukey’s test (D–F).

As we previously reported, BMP‐4 induces differentiation and apoptosis of GICs [20]. Consistently, neurosphere formation by patient‐derived GICs, TGS‐01, in a serum‐free condition, was suppressed by BMP‐4 stimulation (Fig. 1A). BMP‐4 reduced the expression of glioma stem cell marker mRNAs, including *PROM1*, *OLIG2*, and *SOX2*, in TGS‐01 as well as in TGS‐04 cells (Fig. 1B). To address the mechanisms underlying BMP‐induced differentiation of GICs, we screened for genes regulated by BMP‐4 in GICs using an RNA‐seq analysis of TGS‐01 cells treated with or without BMP‐4 [20]. Among the genes induced by BMP‐4 in TGS‐01 cells, we focused our study on the function of the gene encoding PRRX1, because PRRX1 has been reported to play critical roles in normal mouse NSCs [33].

PRRX1 occurs as two isoforms, that is, pmx‐1a and pmx‐1b, that are produced by alternative splicing. The first 199 amino acid residues have identical sequences, but the C‐terminal regions differ between the two isoforms (Fig. 1C). The longer isoform of 245 amino acid residues, pmx‐1b (also known as PRRX1A, encoded by *pmx‐1b*, NM_022716), has an OAR (otp, aristaless, and rax) domain in its C‐terminal region (amino acid residues 200‐245), whereas the shorter isoform of 217 amino acid residues, pmx‐1a (also known as PRRX1B, encoded by *pmx‐1a*, NM_006902), lacks this domain but contains a repressor domain (amino acid residues 200‐217) [34]. qRT‐PCR analysis revealed that both isoforms were upregulated in TGS‐01 and TGS‐04 by BMP‐4 stimulation (Fig. 1D).

The *PROM1* promoter has two highly conserved putative PRRX1‐binding sites (Fig. S1A). ChIP‐qPCR analysis using TGS‐01 cells stably expressing pmx‐1a or pmx‐1b isoforms revealed that both isoforms of PRRX1 bound to the *PROM1* promoter regions containing the two predicted PRRX1‐binding sites (Fig. 1E). Next, luciferase reporter assays were performed to investigate whether PRRX1 regulates *PROM1* promoter activity. Deletion of or mutations in the PRRX1‐binding sites in the *PROM1* promoter showed enhanced promoter activity in TGS‐01 and TGS‐04 cells, which was not significantly affected by BMP‐4 treatment (Fig. 1F, Fig. S1B). Of the two PRRX1‐binding sites, the distal binding site (region −1593 to −1560) was more important than the proximal binding site (region −1033 to −1003). These observations suggest that PRRX1 suppresses the *PROM1* promoter activity by binding to this region.

### 3.2 Silencing *PRRX1* maintains the neurosphere‐forming ability of GICs and induces the acquisition of GIC properties

**Fig. 2:**
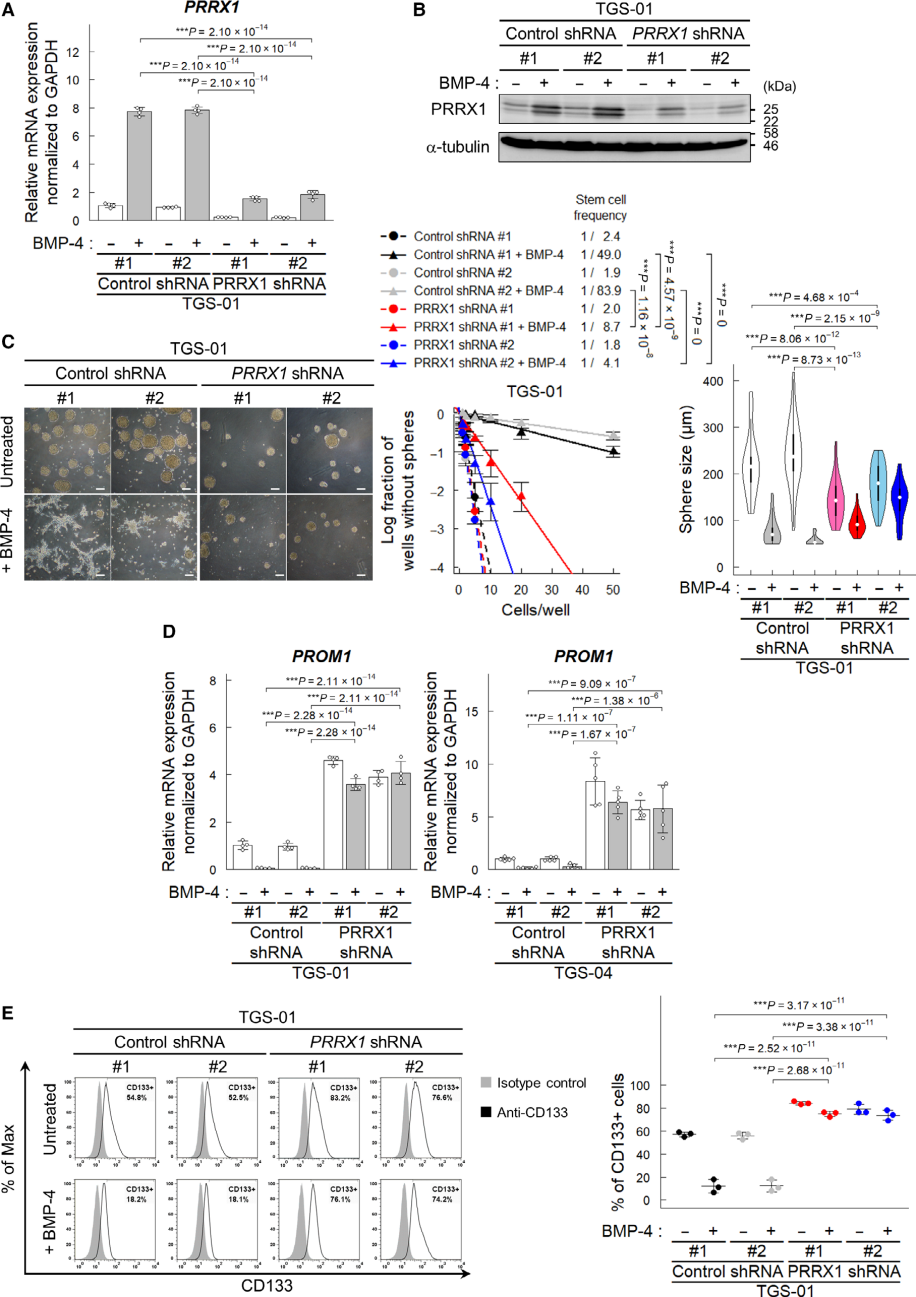
PRRX1 is required for BMP‐induced loss of the CD133‐positive GIC population. TGS‐01 cells expressing shRNA were treated for 72 h with or without BMP‐4 (30 ng·mL^−1^), except for panel (C). (A, B) Knockdown of *PRRX1* mRNA (A) and protein (B) by *PRRX1* shRNA in TGS‐01 cells (*n* = 4 biological replicates in (A)). Representative data of the two independent experiments are shown (B). (C) Regulation of sphere‐forming ability by knockdown of *PRRX1*. Phase‐contrast images showing the morphology of shRNA‐expressing TGS‐01 cells treated with or without BMP‐4 for 5 days under serum‐free condition (left). Scale bars: 200 µm. Sphere‐forming ability was determined by limiting dilution assay (middle, *n* = 3 independent experiments), and the sizes of spheres (cultured at the initial density of 50 cells per well) were determined (right, *n* = 21–48 from 3 independent experiments). Cells were treated with or without BMP‐4 (30 ng·mL^−1^) for 7 days. (D) Regulation of *PROM1* mRNA by silencing of *PRRX1* in TGS‐01 (*n* = 4 biological replicates). (E) CD133 expression on the surface of TGS‐01 cells was evaluated by flow cytometric analysis (*n* = 3 biological replicates). The graphs in panels (A) and (C–E) represent mean ± SD of biological replicates. The *P*‐values were determined by Tukey’s test (A, C–E).

To address whether PRRX1 plays a role in maintaining stem cell‐like properties of GICs, we silenced *PRRX1* expression in GICs and examined their neurosphere formation. Lentiviral‐mediated shRNA reduced the expressions of PRRX1 mRNA and protein in TGS‐01 and TGS‐04 cells (Fig. 2A,B and Fig. S2A,B). While the neurosphere formation of TGS‐01 cells expressing nontargeting control shRNA was suppressed by BMP‐4 stimulation, TGS‐01 cells expressing *PRRX1* shRNA maintained the ability to form neurospheres even in the presence of BMP‐4, although the size of spheres decreased by knockdown of *PRRX1* in the absence of BMP‐4 stimulation (Fig. 2C). In TGS‐04 cells, knockdown of *PRRX1* resulted in the increased ability of GICs to form neurospheres in the presence and absence of BMP‐4; similar to TGS‐01 cells, the size of spheres decreased by knockdown of *PRRX1* in the absence of BMP‐4 stimulation in TGS‐04 cells (Fig. S2C).

We next examined the effect of *PRRX1* knockdown on the expression levels of glioma stem cell markers. The expression of *PROM1* mRNA encoding CD133 was upregulated in GICs expressing *PRRX1* shRNA compared to those expressing control shRNA (Fig. 2D). In contrast, silencing of *PRRX1* in GICs only showed weak or nonsignificant effects on *OLIG2* or *SOX2* expression, suggesting that the effects of BMP‐4‐induced PRRX1 on the expression of stem cell markers are specific to some markers, such as *PROM1* (Fig. S2D,E). Consistent with the increase in *PROM1* mRNA expression, flow cytometric analysis revealed that the CD133‐positive population was increased by *PRRX1* silencing in TGS‐01 and TGS‐04 cells even in the presence of BMP‐4 (Fig. 2E and Fig. S2F).

**Fig. 3:**
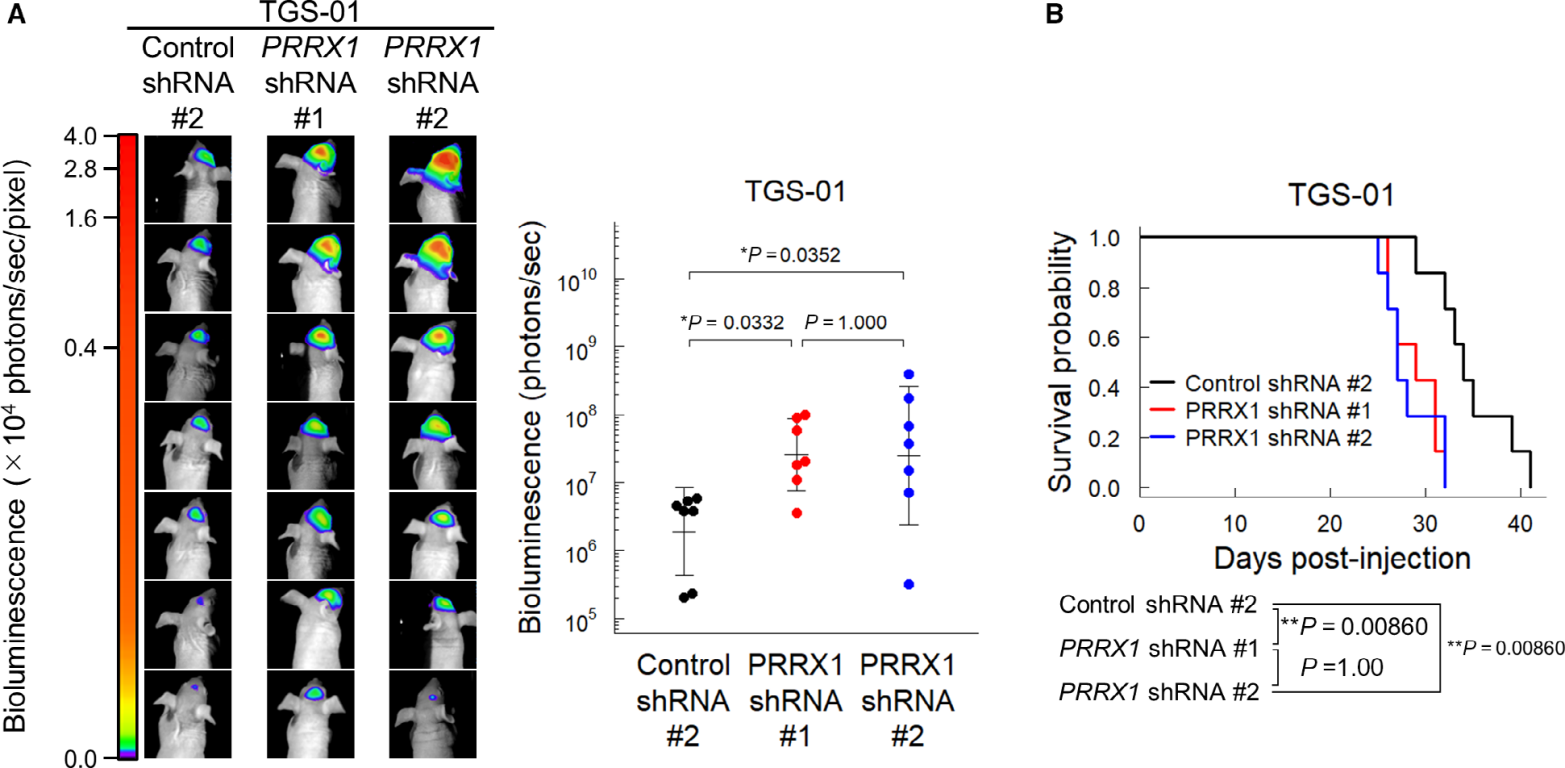
Effect of *PRRX1* knockdown in TGS‐01 cells *in vivo*. Intracranial proliferation of GICs expressing *firefly* luciferase and control or *PRRX1* shRNA (*n* = 7 mice for each group). (A) *In vivo* bioluminescent imaging analysis was performed 2 weeks after intracranial injection. (B) Kaplan–Meier survival curves of mice injected with TGS‐01 cells expressing control or *PRRX1* shRNA. The graph represents mean ± SD of biological replicates (A). The *P*‐values were determined by Tukey’s test (A) or by two‐tailed log‐rank test and then adjusted by Bonferroni correction (B).

To address whether PRRX1 expression in GICs affects their tumorigenic activity, GICs expressing *PRRX1* shRNA, or nontargeting control shRNA, together with *firefly* luciferase were injected intracranially into nude mice. *In vivo* bioluminescent imaging of orthotopic tumors revealed that silencing *PRRX1* in TGS‐01 cells accelerated tumor growth (Fig. 3A) and consistently shortened the survival of the mice (Fig. 3B).

### 3.3 The PRRX1 pmx‐1b isoform plays an essential role in impairment of GIC properties

**Fig. 4:**
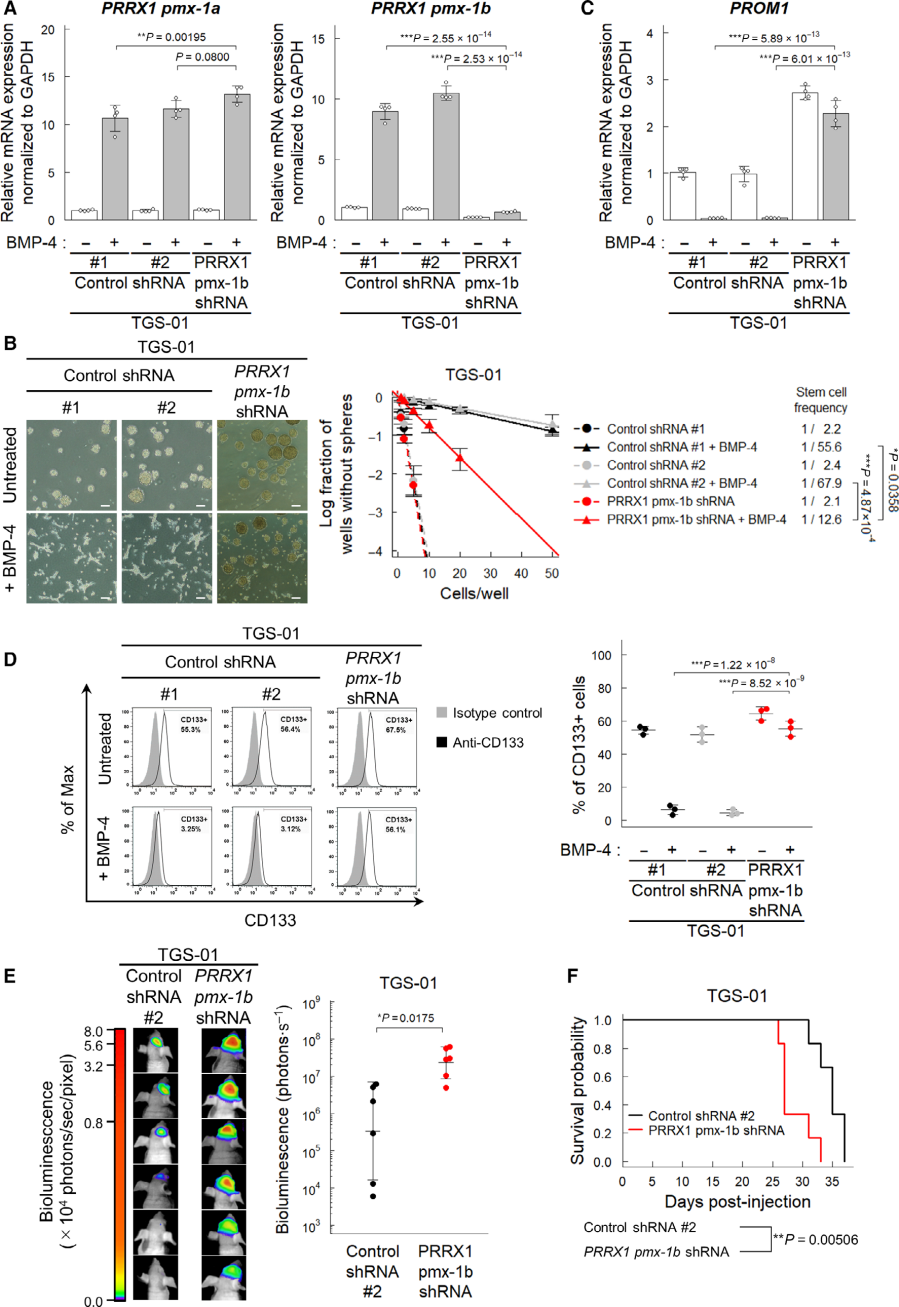
The PRRX1 pmx‐1b isoform is important for the decrease in the CD133‐positive population of GICs. TGS‐01 cells were treated with or without BMP‐4 (30 ng·mL^−1^) for 72 h, except for panel (B). (A) Knockdown of *PRRX1 pmx‐1b* by a shRNA specific against the pmx‐1b isoform in TGS‐01 cells (*n* = 4 biological replicates). (B) Phase‐contrast images showing the morphology of shRNA‐expressing TGS‐01 cells treated with or without BMP‐4 (30 ng·mL^−1^) for 5 days under the serum‐free condition (left). Scale bars: 200 µm. Sphere‐forming ability was determined by limiting dilution assay (right, *n* = 3 independent experiments). Cells were treated with or without BMP‐4 (30 ng·mL^−1^) for 7 days. (C) Upregulation of *PROM1* mRNA by treatment with shRNA against the *PRRX1 pmx‐1b* isoform in TGS‐01 cells (*n* = 4 biological replicates). (D) Surface expression of CD133 in TGS‐01 cells evaluated by flow cytometric analysis (*n* = 3 biological replicates). (E, F) Intracranial proliferation of GICs expressing *firefly* luciferase and control or *pmx‐1b* shRNA (*n* = 6 mice for each group). The *in vivo* bioluminescent imaging analysis was performed 2 weeks after the intracranial injection (E). Kaplan–Meier survival curves of mice injected with TGS‐01 cells expressing control or *pmx‐1b* shRNA (F). The graphs in panels (A–E) represent mean ± SD of biological replicates. The *P*‐values were determined by Tukey’s test (A–D), Student’s *t*‐test (E), or two‐tailed log‐rank test (F).

Distinct functions have been reported for the two isoforms of PRRX1 in certain cancers, as well as in human oligodendrocyte progenitor cells (hOPCs) [34, 35, 36, 37]. To investigate whether the isoforms have functional differences in GICs, the *pmx‐1b* isoform alone was knocked down in TGS‐01 and TGS‐04 cells by shRNA (Fig. 4A and Fig. S3A). As observed in GICs expressing shRNA against both isoforms of PRRX1, GICs expressing the *pmx‐1b* shRNA formed neurospheres even in the presence of BMP‐4 with significantly increased stem cell frequency (Fig. 4B and Fig. S3B). Moreover, silencing the *pmx‐1b* isoform induced *PROM1* expression and increased the CD133‐positive population even in the presence of BMP‐4 (Fig. 4C,D and Fig. S3C,D). To clarify the effect of the pmx‐1b isoform on *in vivo* tumorigenicity, TGS‐01 cells expressing shRNA against the pmx‐1b isoform were injected intracranially into nude mice. In a similar way to the silencing of *PRRX1*, knockdown of the pmx‐1b isoform alone in TGS‐01 cells enhanced their tumorigenic activity and shortened the survival of the mice (Fig. 4E,F). These results suggest that the pmx‐1b isoform of PRRX1 is essential for BMP‐induced differentiation of GICs.

### 3.4 The PRRX1 pmx‐1b isoform depletes neurosphere‐forming ability of GICs and induces loss of GIC properties

**Fig. 5:**
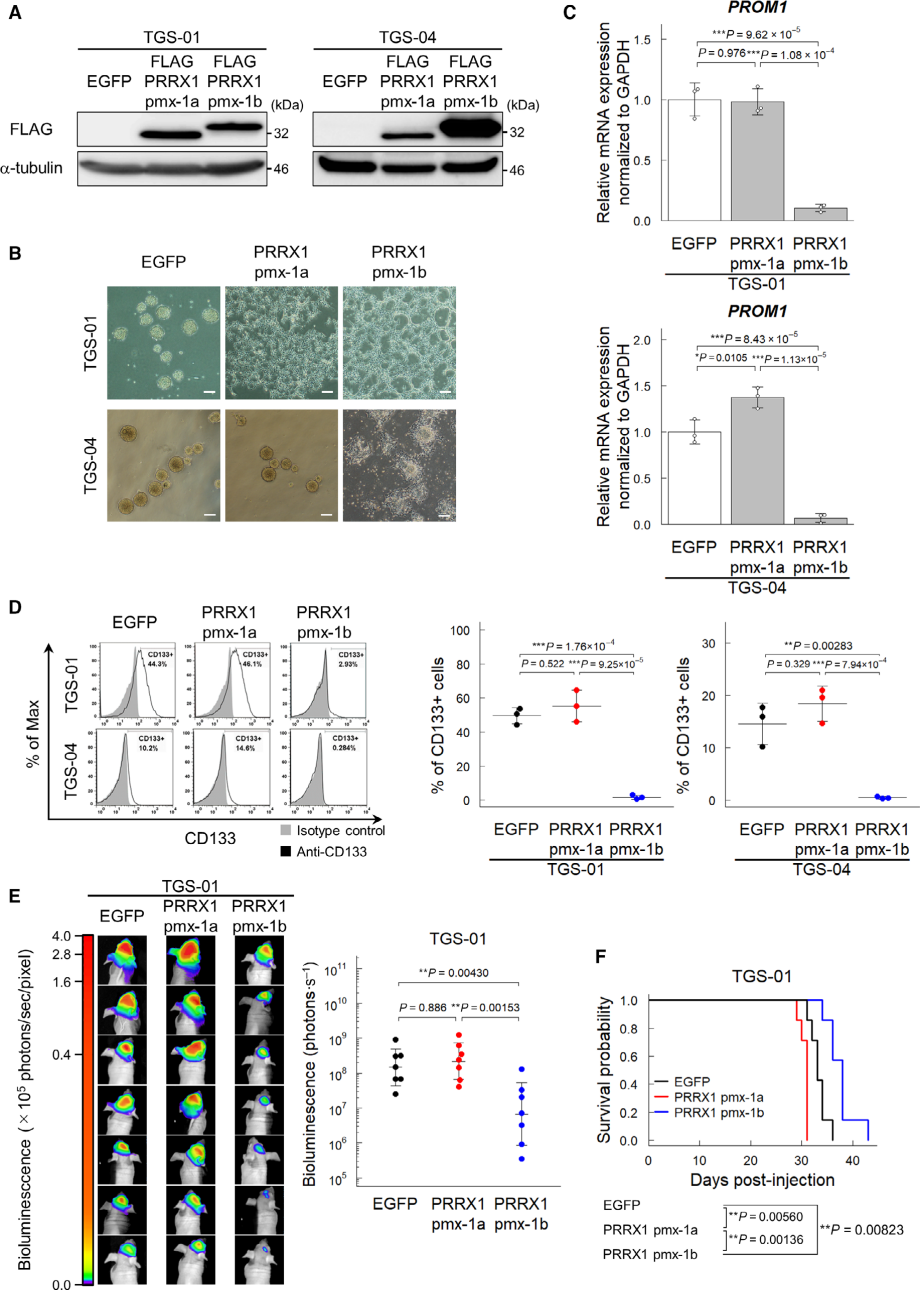
The PRRX1 pmx‐1b isoform reduces the CD133‐positive population of GICs. Cells were treated with or without BMP‐4 (30 ng·mL^−1^) for 72 h, except for panel (B). (A) Immunoblot analysis of enforced expression of FLAG‐tagged pmx‐1a or pmx‐1b in TGS‐01 and TGS‐04 cells. (B) Phase‐contrast images showing the morphology of EGFP‐ and FLAG‐pmx‐1a‐ or FLAG‐pmx‐1b‐expressing TGS‐01 and TGS‐04 cells treated with or without BMP‐4 (30 ng·mL^−1^) for 5 days under serum‐free condition. Scale bars: 200 µm. (C) Downregulation of *PROM1* mRNA by ectopic expression of the PRRX1 pmx‐1b isoform in TGS‐01 and TGS‐04 cells (*n* = 3 biological replicates). (D) Surface expression of CD133 in TGS‐01 and TGS‐04 cells evaluated by flow cytometric analysis. Ectopic expression of the PRRX1 pmx‐1b decreased the CD133‐positive population (*n* = 3 biological replicates). (E, F) *In vivo* tumorigenic activity of GICs expressing *firefly* luciferase and EGFP, pmx‐1a, or pmx‐1b (*n* = 7 mice for each group). The *in* *vivo* bioluminescent imaging analysis was performed 2 weeks after intracranial injection (E). Kaplan–Meier survival curves of mice injected with TGS‐01 cells expressing EGFP, pmx‐1a, or pmx‐1b (F). The graphs in panels (C–E) represent mean ± SD of biological replicates. The *P*‐values were determined by Tukey’s test (C–E). In panel (F), the *P*‐value was determined by two‐tailed log‐rank test and then adjusted by Bonferroni correction.

To test whether PRRX1 induction is sufficient for loss of stem cell‐like properties in GICs, we examined the effect of ectopic expression of PRRX1 on neurosphere formation and glioma stem cell marker expression. Enforced expression of either pmx‐1a or pmx‐1b suppressed the neurosphere‐forming ability of TGS‐01 cells, whereas only pmx‐1b, but not pmx‐1a, suppressed the neurosphere‐forming ability of TGS‐04 cells (Fig. 5A,B). Inhibition of sphere formation of TGS‐01 cells by the ectopic expression of pmx‐1a may not be due to loss of the stem cell‐like phenotype, because only the pmx‐1b isoform, but not the pmx‐1a isoform, drastically reduced the expression levels of *PROM1* in both TGS‐01 cells and TGS‐04 cells (Fig. 5C). We also confirmed that the ectopic expression of only pmx‐1b, but not pmx‐1a, depleted the CD133‐positive populations of TGS‐01 and TGS‐04 cells (Fig. 5D).

To examine the effect of expression of PRRX1 isoforms on tumorigenicity, TGS‐01 cells ectopically expressing pmx‐1a, pmx‐1b, or EGFP, together with *firefly* luciferase, were injected intracranially into nude mice. Ectopic expression of the pmx‐1b isoform suppressed tumor growth and significantly prolonged the survival of the mice, while the expression of the pmx‐1a isoform shortened the life span of the mice (Fig. 5E,F). These findings indicate that the PRRX1 pmx‐1b isoform plays a crucial role in BMP‐induced loss of stem cell‐like properties in GICs.

To investigate the generality of the effects of PRRX1 on *PROM1* expression, we used two other GICs, U3005MG and U3024MG cells, that were obtained from the HGCC resource [30]. BMP‐4 induced the pmx‐1a and pmx‐1b isoforms of PRRX1, and silencing of *PRRX1* or *pmx‐1b* strongly induced *PROM1* expression in U3005MG and U3024MG cells as well as in TGS‐01 and TGS‐04 cells (Fig. S4A,B). Moreover, ectopic expression of only pmx‐1b, but not pmx‐1a, reduced the expression levels of *PROM1* in U3005MG and U3024MG cells (Fig. S4C).

### 3.5 DNMT3A is involved in the reduction of CD133 expression after BMP‐4 stimulation

**Fig. 6:**
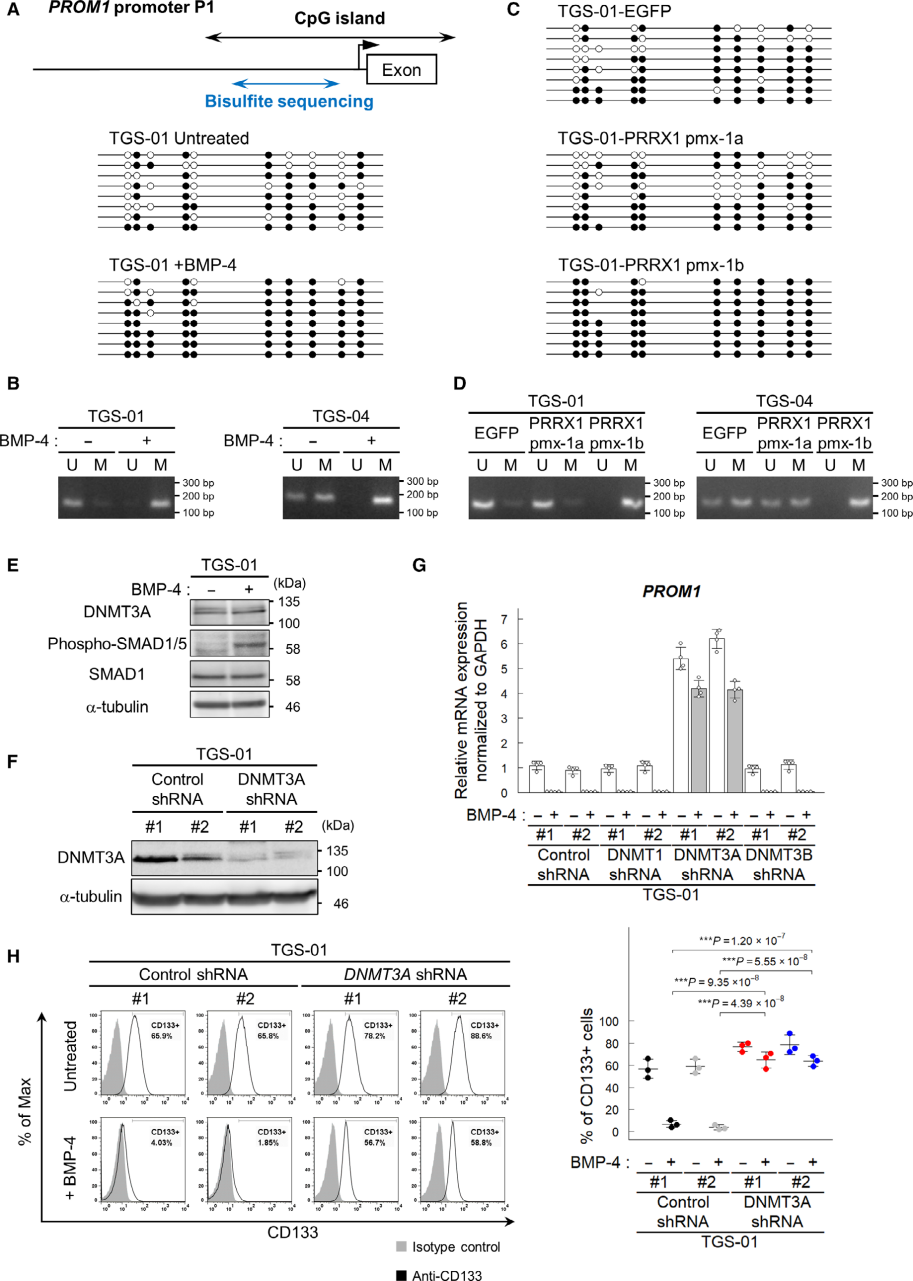
DNMT3A is involved in the reduction of CD133 expression by BMP signaling. (A, B) DNA methylation of the *PROM1* P1 promoter region by BMP‐4. Cells were treated with BMP‐4 (30 ng·mL^−1^) for 72 h. U, unmethylated and M, methylated. (C, D) DNA methylation of the *PROM1* P1 promoter region by ectopic expression of pmx‐1a or pmx‐1b. (E, F) Immunoblot analysis of the expression of DNMT3A protein in TGS‐01 cells treated with or without BMP‐4 (30 ng·mL^−1^) for 72 h (E) and with or without downregulation of DNMT3A by shRNA in TGS‐01 cells (F). (G) Screening for DNA methyltransferases involved in the expression of *PROM1*. DNA methyltransferases (*DNMT1*, *DNMT3A*, and *DNMT3B*) were knocked down by shRNAs in TGS‐01 cells, and expression levels of *PROM1* (encoding CD133) were quantified by real‐time qRT‐PCR. The cells were treated with or without BMP‐4 (30 ng·mL^−1^) for 72 h (*n* = 4 biological replicates). (H) Surface expression of CD133 evaluated by flow cytometric analysis. TGS‐01 cells expressing control or *DNMT3A* shRNA were treated for 72 h with or without BMP‐4 (30 ng·mL^−1^). The graphs in panels (G, H) represent mean ± SD of biological replicates. The *P*‐values were determined by Tukey’s test.

A CpG island is present in the vicinity of PRRX1‐binding sites in the *PROM1* promoter P1 region. Although the CpG sites in the *PROM1* promoter P1 region are hypomethylated in CD133‐positive glioblastoma cells, they are highly methylated in CD133‐negative glioblastoma and in normal neural tissues [38]. Since PRRX1 directly regulates CD133 expression in GICs, we examined whether BMP‐4 stimulation affects DNA methylation in the *PROM1* promoter. BMP‐4 increased the levels of DNA methylation in the *PROM1* promoter P1 region in TGS‐01 and TGS‐04 cells (Fig. 6A,B). Moreover, the ectopic expression of pmx‐1b, but not pmx‐1a, increased DNA methylation levels in TGS‐01 and TGS‐04 cells (Fig. 6C,D).

To examine which DNMTs are involved in BMP‐induced DNA methylation in the *PROM1* promoter P1 region in GICs, each of the three DNMTs was knocked down by shRNA in TGS‐01 and TGS‐04 cells (Fig. 6E,F, Fig. S5A–D). BMP‐4 stimulation did not strongly affect DNMT3A expression in TGS‐01 and TGS‐04 cells (Fig. 6E and Fig. S5A–D). Of the three DNMTs in mammals, silencing the expression of *DNMT3A*, but not that of *DNMT1* or *DNMT3B*, induced the expression of *PROM1* (Fig. 6G and Fig. S5E). The downregulation of DNMT3A expression by shRNA resulted in an increase in the CD133‐positive population even in the presence of BMP‐4 stimulation (Fig. 6H and Fig. S5F). These findings suggest that DNMT3A is critically involved in the decrease in the CD133‐positive population.

### 3.6 The PRRX1 pmx‐1b isoform cooperates with DNMT3A to downregulate CD133 expression

**Fig. 7:**
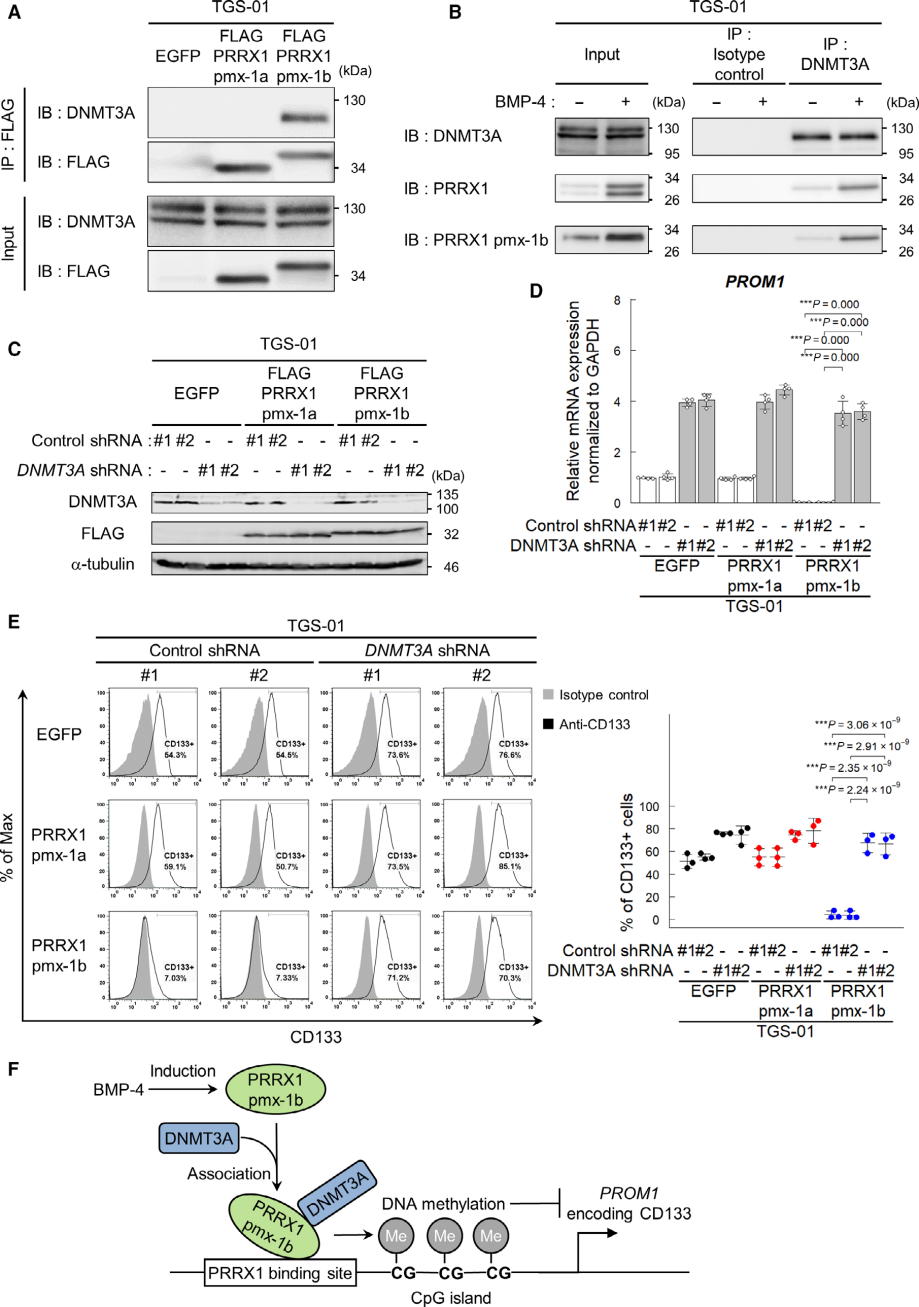
DNMT3A is required for downregulation of CD133 expression by PRRX1 pmx‐1b isoform. (A, B) Immunoblot analysis to examine the physical interaction between DNMT3A and PRRX1. In TGS‐01 cells expressing FLAG‐tagged PRRX1, DNMT3A was co‐immunoprecipitated with FLAG‐tagged PRRX1 pmx‐1b, but not with FLAG‐tagged pmx‐1a (A). In TGS‐01 cells treated for 72 h with or without BMP‐4 (30 ng·mL^−1^), endogenous PRRX1 pmx‐1b was co‐immunoprecipitated with DNMT3A (B). Representative data of the three independent experiments are shown (A, B). (C–E) DNMT3A shRNA and FLAG‐tagged PRRX1 were transduced into TGS‐01. Immunoblot analysis showing the expression of DNMT3A shRNA and FLAG‐tagged PRRX1 in TGS‐01 cells (C). Expression of *PROM1* in TGS‐01 cells expressing DNMT3A shRNA and PRRX1 (*n* = 4 biological replicates) (D). CD133 expression on cell surface evaluated by flow cytometric analysis in TGS‐01 cells (*n* = 3 biological replicates) (E). The graphs in panels (D, E) represent mean ± SD of biological replicates. The *P*‐values were determined by Tukey’s test (D, E). (F) A scheme showing the regulation of CD133 expression by PRRX1 pmx‐1b and DNMT3A regulated by BMP stimulation. Of the two splice isoforms, only the longer isoform of PRRX1, pmx‐1b, induces the differentiation of GICs. Through functional cooperation with DNMT3A, pmx‐1b induces the promoter methylation of the key stem cell marker gene *PROM1* by recruitment of DNMT3A and suppresses the CD133‐positive GIC population.

The pmx‐1a and pmx‐1b isoforms of PRRX1 can bind to the *PROM1* promoter P1 region (Fig. 1E). However, these two isoforms, which have different C‐terminal regions, showed differential effects on the transcription of *PROM1* (Fig. 4C, Figs S3C and S4C). We thus examined whether the two isoforms of PRRX1 physically interact with DNMT3A. Immunoprecipitation of FLAG‐tagged PRRX1 followed by immunoblotting with a DNMT3A antibody revealed that pmx‐1b, but not pmx‐1a, interacted with DNMT3A (Fig. 7A). Moreover, endogenous interaction between the DNMT3A and pmx‐1b proteins was observed in the presence and absence of BMP‐4 stimulation in TGS‐01 cells (Fig. 7B).

To study how pmx‐1b and DNMT3A cooperatively regulate the transcription of *PROM1*, *DNMT3A* shRNA and *PRRX1* were transduced in GICs (Fig. 7C and Fig. S6A). Silencing of *DNMT3A* increased the expression of *PROM1* mRNA and CD133‐positive populations of TGS‐01 and TGS‐04 cells (Fig. 6G,H and Fig. S5E,F). Although the ectopic expression of pmx‐1b, but not of pmx‐1a, decreased *PROM1* mRNA expression and the CD133‐positive population, the effects of pmx‐1b were drastically attenuated by silencing of *DNMT3A* (Fig. 7D,E and Fig. S6B,C). These findings reveal that, upon interaction with the pmx‐1b isoform, DNMT3A decreases the CD133‐positive population of GICs through epigenetic regulation of the *PROM1* promoter by DNMT3A (Fig. 7F).

## 4 Discussion

BMPs play pivotal roles in the growth, differentiation, and function of normal neural cells, and BMP signaling regulates the differentiation of normal NSCs [39, 40]. Although many studies have shown that BMP signaling induces growth arrest, apoptosis, and differentiation of GICs [23], GICs show heterogeneous responses to BMPs, and certain populations of GICs escape the differentiation‐inducing effects of BMPs [25]. Several BMP‐induced molecules have been shown to be involved in the induction of cell cycle arrest, apoptosis, and cell differentiation of GICs [18, 19, 20, 26, 27], but less is known about the BMP target molecules that are involved in the regulation of the stem cell‐like properties of GICs.

Through RNA‐seq analysis using GICs treated with or without BMP‐4, we identified *PRRX1* as a BMP target gene in human GICs. PRRX1 is a homeobox transcription factor containing the paired‐type DNA‐binding homeodomain, which is essential for normal development. PRRX1 is widely expressed in various cells, including undifferentiated mesenchymal cells, and *Prrx1*‐deficient mice show perinatal death with skeletal malformations [41]. In the development of the nervous system, PRRX1 is involved in the maintenance of self‐renewal of NSCs in cooperation with SOX2, and depletion of *Prrx1* expression in cultured adult mouse NSCs results in attenuation of their self‐renewal ability [33]. Moreover, the knockdown of *Prrx1* expression in NSCs induces the expression of genes involved in neuronal and glial differentiation.

Although PRRX1 plays a critical role in the maintenance of the stem cell‐like properties of normal NSCs [33], it also suppresses proliferation and migration of some neuronal progenitor cells. PRRX1 has been reported to upregulate the expression of cyclin dependent kinase inhibitors, including p21^WAF1/CIP1^ and p15^INK4B^, and induce the cell cycle arrest of hOPCs [37]. Interestingly, both isoforms of PRRX1 suppressed hOPC proliferation, whereas only pmx‐1b, but not pmx‐1a, reduced cell migration of hOPCs. In addition to human GICs, BMP treatment increased both pmx‐1a and pmx‐1b in hOPCs.

In the present study, we showed that silencing of the *PRRX1* expression maintained the stem cell‐like phenotype of GICs. PRRX1 bound to the *PROM1* promoter and suppressed the expression of CD133. In contrast, predicted PRRX1‐binding sites were not detected in the promoter regions of *OLIG2* and *SOX2* (data not shown). Thus, the effects of PRRX1 on normal and malignant neural stem or progenitor cells may thus be cell‐ and context‐dependent, possibly through the interaction with other transcription factors. To confirm that the effects of PRRX1 on *PROM1* expression are not limited to TGS‐01 and TGS‐04 cells, we also used U3005MG and U3024MG cells obtained from the HGCC resource, which are maintained under serum‐free conditions to preserve the GIC characteristics [30] and respond to BMP‐4 stimulation (data not shown). Consistent with TGS‐01 and TGS‐04 cells, the PRRX1 pmx‐1b isoform suppressed *PROM1* expression in U3005MG and U3024MG cells (Fig. S4).

CD133‐positive cells in human glioblastoma have two key properties of cancer‐initiating cells, that is, the ability to self‐renew and to recapitulate tumor heterogeneity through cell differentiation [42]. Some reports suggested that CD133 is not a universal marker of GICs, and instances of CD133‐negative cells exhibiting similar self‐renewal properties as CD133‐positive cells have also been reported [6, 43, 44, 45, 46]. However, knockdown of *PROM1*/CD133 leads to the disruption of self‐renewal and tumorigenic abilities of neurosphere‐derived human glioblastoma cells, and the re‐expression of CD133 restores the stem cell‐like phenotype in *PROM1*/CD133‐silenced glioblastoma cells, suggesting that CD133 plays an important role in the maintenance of stem cell‐like properties of GICs [11].

Yan *et al*. reported that a CD133‐related gene expression signature obtained by gene expression profiling experiments using DNA microarrays resembles human embryonic stem cells and glioblastoma stem cells. Moreover, the CD133 gene expression signature identifies aggressive subpopulations of glioblastoma [47]. Phosphorylation of Tyr828 in the cytoplasmic domain of CD133 mediates interaction with the p85 subunit of phosphoinositide 3’‐kinase (PI3K), leading to activation of the PI3K‐Akt pathway in GICs [48]. Thus, silencing *PROM1*/CD133 expression suppresses the activity of the PI3K‐Akt pathway, resulting in attenuation of the self‐renewal and tumorigenicity of GICs. In agreement with these findings, our data demonstrated that decreased CD133 expression by the knockdown of *PRRX1* expression resulted in loss of neurosphere‐forming ability and tumorigenic activity of GICs.

The two major isoforms of PRRX1, pmx‐1a (PRRX1B) and pmx‐1b (PRRX1A), are generated by alternative splicing and have distinct functions [35, 36, 37]. The C‐terminal region of pmx‐1b containing the OAR domain can mask transcription activation, whereas the C‐terminal of pmx‐1a contains a repression domain [34]. During the development and dissemination of pancreatic ductal adenocarcinoma, pmx‐1b stimulates mesenchymal–epithelial transition, tumor differentiation, and metastatic colonization of the tumor, whereas pmx‐1a facilitates epithelial–mesenchymal transition, tumor de‐differentiation, and invasion of tumor cells [36].

In the present study, we showed that of the two major isoforms of PRRX1, pmx‐1b plays a major role in the induction of GIC differentiation. Both pmx‐1a and pmx‐1b are expressed in the GICs used in the present study, and BMP‐4 stimulation induced both isoforms (Fig. 1D). Silencing of *pmx‐1b* alone mimicked the effect of knockdown of both isoforms (Fig. 4, Figs S3 and S4A,B). On the other hand, the enforced expression of pmx‐1a failed to decrease the population of CD133‐positive GICs (Fig. 5D), and inoculation of TGS‐01 cells expressing pmx‐1a resulted in shorter survival of mice than inoculation with control GICs expressing EGFP (Fig. 5F).

Knockdown of *PRRX1* was reported to attenuate the invasiveness and neurosphere‐forming ability of glioblastoma cell lines, including T98 and U251MG, in a serum‐containing condition [49]. PRRX1 was also reported to transactivate dopamine D2 receptor in GICs cultured in serum‐free conditions and maintain the tumorigenic activity of GICs *in vivo* [50]. Functions of the two PRRX1 isoforms were not investigated in these studies [49, 50]. In the GICs used in the present study, expression of dopamine D2 receptor was not induced by BMP signaling nor suppressed by *PRRX1* knockdown (data not shown). PRRX1 may exhibit different functions in GICs, possibly depending on cell culture conditions, cell differentiation status, and balance of the expression of the two PRRX1 isoforms.

DNA methylation is a highly regulated process in normal cells, whereas methylation profiles are drastically altered in various cancer cells [51]. Compared to normal cells, cancer cells show global hypomethylation of genomic DNA and specific hypermethylation of the promoters of various tumor suppressor genes. DNA methylation is induced by three different DNA methyltransferases in mammals; DNMT3A and DNMT3B are involved in the *de novo* methylation of CpG islands, whereas DNMT1 is mainly responsible for the maintenance of established DNA methylation patterns. Although DNA methyltransferases play critical roles in differentiation of various stem cells, including embryonic stem cells, neural stem cells, and hematopoietic stem cells [52, 53], it is not fully understood how they are recruited to chromatin and induce methylation of particular sites. Mutations in the *DNMT3A* gene are often observed in hematological malignancies, but less frequently in other cancers. In contrast, aberrant expression of DNMTs, for example, high expression of DNMT1 and DNMT3B [54] and low expression of DNMT3A [55], has been reported in glioblastoma. DNMT3A was shown to play a crucial role in the proliferation and differentiation of mouse NSCs [53, 56]; however, little is known about how DNMTs regulate the differentiation of glioblastoma cells.

In the present study, we showed that, among the three DNA methyltransferases, the silencing of *DNMT3A* maintained the expression of *PROM1* and the number of CD133‐positive glioblastoma cells, even in the presence of BMP‐4 (Fig. 6 and Fig. S5). Co‐immunoprecipitation analysis demonstrated that the pmx‐1b, but not the pmx‐1a isoform, interacted with DNMT3A (Fig. 7A,B). The functional interaction between pmx‐1b and DNMT3A may thus facilitate DNA methylation of the *PROM1* gene promoter. DNMT1 and DNMT3A have been shown to interact with the transcription factor ZEB1 and HMGA2, respectively, and maintain the DNA methylation of the *CDH1* promoter in normal and malignant breast epithelial cells during epithelial–mesenchymal transition [57, 58]. Together, these findings suggest that DNA methylation may be induced only in certain genomic regions through recruitment of DNA methyltransferases by DNA‐binding proteins.

Using the public database TCGA, we analyzed the expression of genes studied in the present study, including *PRRX1*, *PROM1*, and *DNMT3A*, to investigate possible correlations between the expression of these genes, the brain tumor grade, and patient prognosis. However, we failed to find any clear tendency of correlations by these analyses. This may be because the populations of GICs are small, and their characteristics may thus not be reflected in the gene expression of the brain tumors as a whole. In addition, the mechanism proposed in the present study may be primarily related to CD133‐positive stem‐like populations.

Epigenetic gene silencing is induced by modifications of histones, DNA methylation, and other components of chromatin. Histone modification by polycomb repressive complex 2, through its catalytic subunit EZH2, induces the epigenetic silencing of the *BMPR1B/ALK6* gene, leading to escape from the BMP‐induced differentiation of glioblastoma cells [17]. Our findings reveal another important mechanism of the epigenetic regulation of GIC differentiation by BMPs. Since recruitment of DNMT3A by the pmx‐1b isoform of PRRX1 leads to GIC differentiation through the epigenetic regulation of *PROM1* gene expression, pmx‐1b and DNMT3A are potentially interesting targets for the treatment of glioblastoma.

## 5 Conclusion

The present study reveals a mechanism of differentiation induction of GICs by BMPs. Through recruitment of DNMT3A to the *PROM1* promoter, the longer splice isoform of PRRX1, induced by BMP signaling, enhances the promoter methylation of the key stem cell marker gene *PROM1* and drives differentiation of GICs.

## Conflict of interest

Satoshi Sakai is currently an employee of Asahi Kasei Pharma Corporation. The other authors declare no competing or financial interests.

## Author contributions

RT, KM, and C‐HH designed the study and wrote the manuscript. RT performed most of the experiments. TT, NT, and BW provided essential experimental materials. TT, CI, AK, SS, ER, DK, MM, and BW participated in experimental design and data analyses. All authors participated in writing, review, and revision of the manuscript.

### Peer Review

The peer review history for this article is available at https://publons.com/publon/10.1002/1878‐0261.13051.

## Supporting information


**Fig. S1**. PRRX1 binds to the *PROM1* promoter and negatively modulates its promoter activity.
**Fig. S2**. PRRX1 is required for BMP‐induced loss of the CD133‐positive GIC population.
**Fig. S3**. The PRRX1 pmx‐1b isoform is important for the decrease in the CD133‐positive population of GICs.
**Fig. S4**. Upregulation of *PROM1* mRNA by treatment with shRNA for *PRRX1* or the *pmx‐1b* isoform in U3005MG and U3024MG cells.
**Fig. S5**. DNMT3A is involved in the reduction of CD133 expression by BMP signaling.
**Fig. S6**. DNMT3A is required for the PRRX1 pmx‐1b isoform to downregulate CD133 expression.

**Table S1**. Characteristics of glioblastoma cells used in the present study.
**Table S2**. Primer sets for real‐time qPCR.
**Table S3**. Target sequences of shRNA.
**Table S4**. Oligonucleotides used for mutagenesis of the PRRX1‐binding sites.
**Table S5**. Primers used for ChIP‐qPCR.
**Table S6**. Primer sets used for bisulfite sequencing.
**Table S7**. Primer sets used for methylation‐specific PCR.

## Data Availability

The data that support the findings of this study are available from the corresponding author upon reasonable request.
