# Supplementary material for: PRRX1 induced by BMP signaling decreases tumorigenesis by epigenetically regulating glioma‐initiating cell properties via DNA methyltransferase 3A
Source: Mol Oncol. 2021 Jul 16;16(1):269–88. doi: 10.1002/1878-0261.13051 (PMC8732353; doi:10.1002/1878-0261.13051)

Supplementary Figure S1.

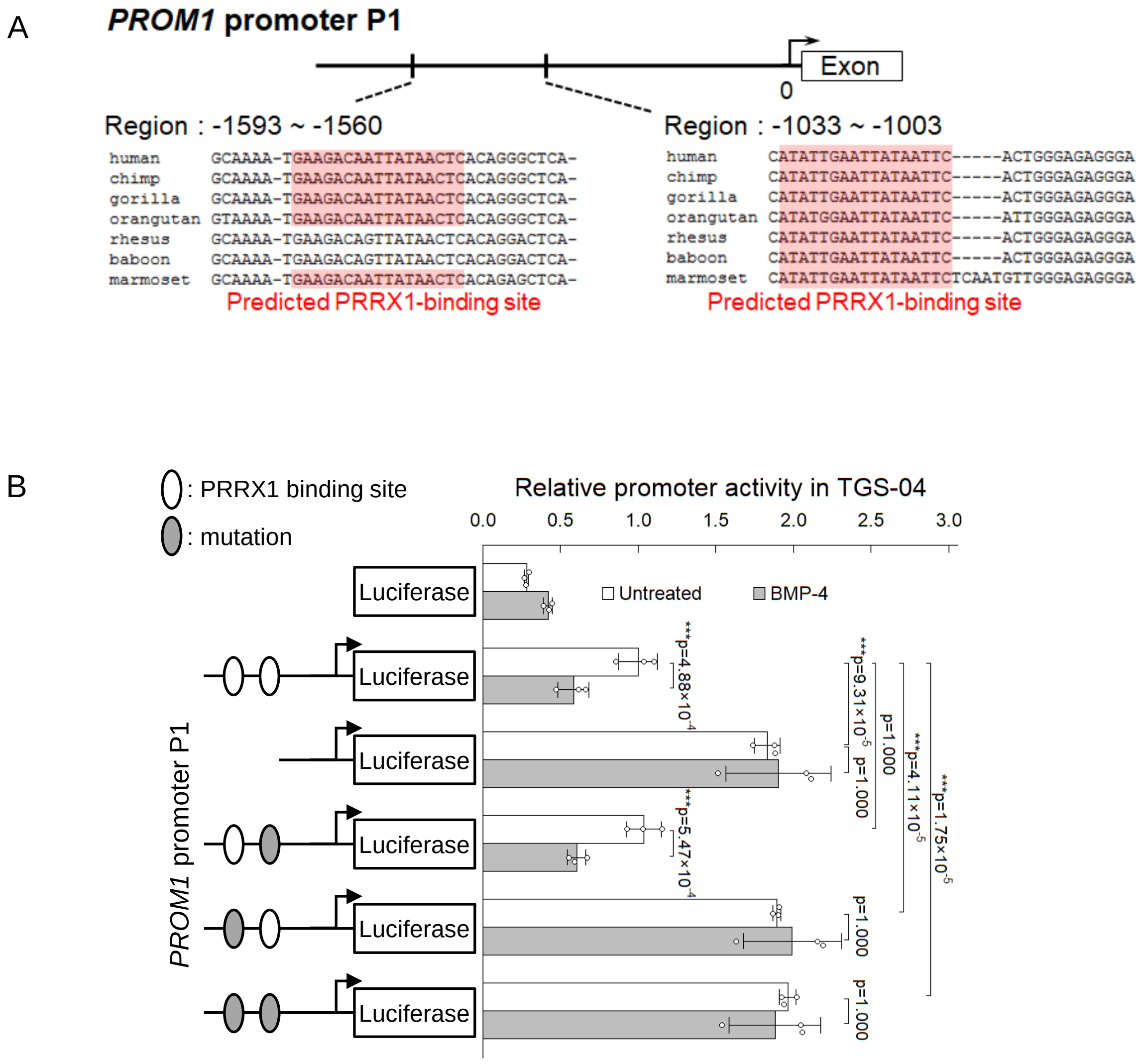

Supplementary Figure S2.

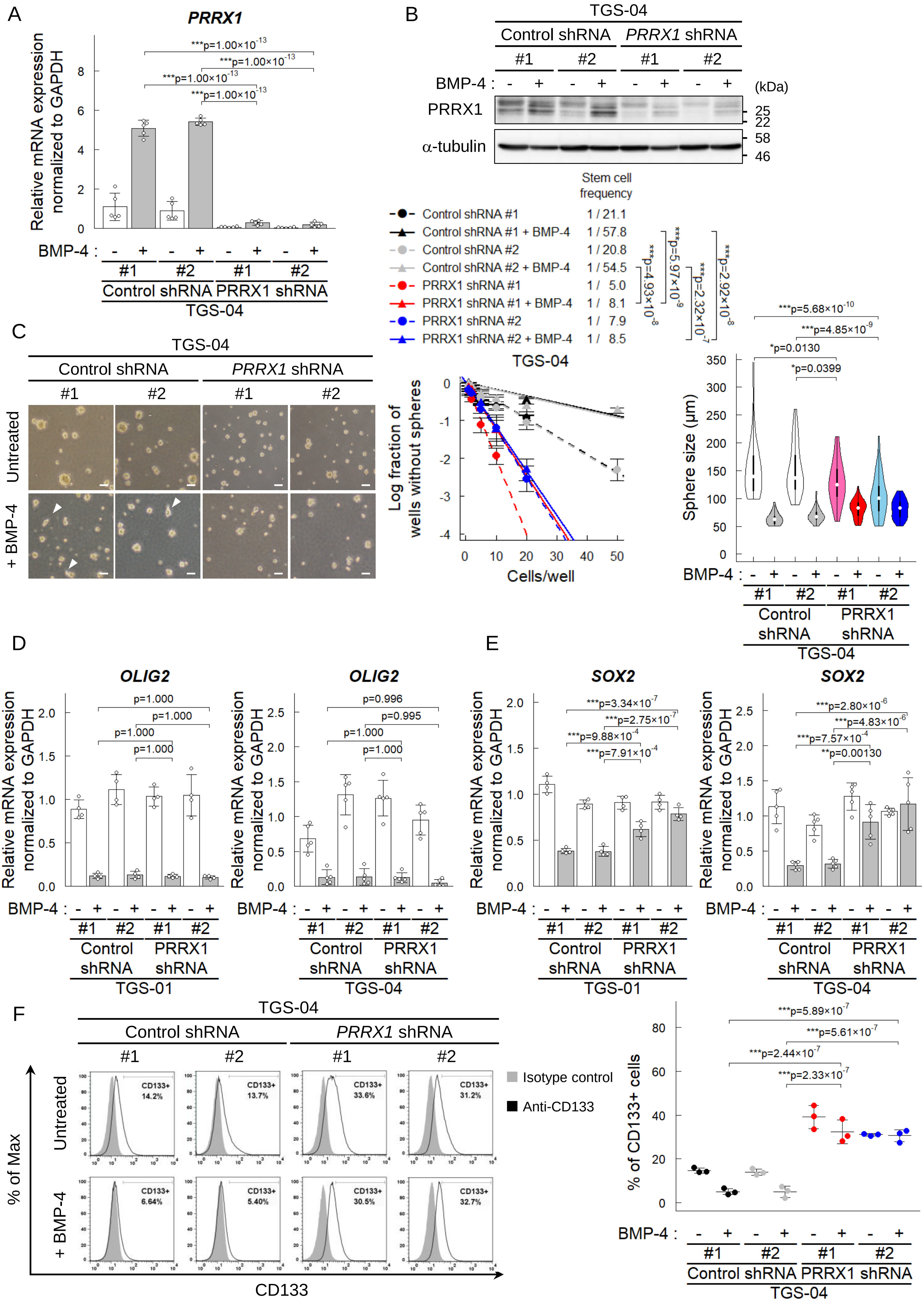

Supplementary Figure S3.

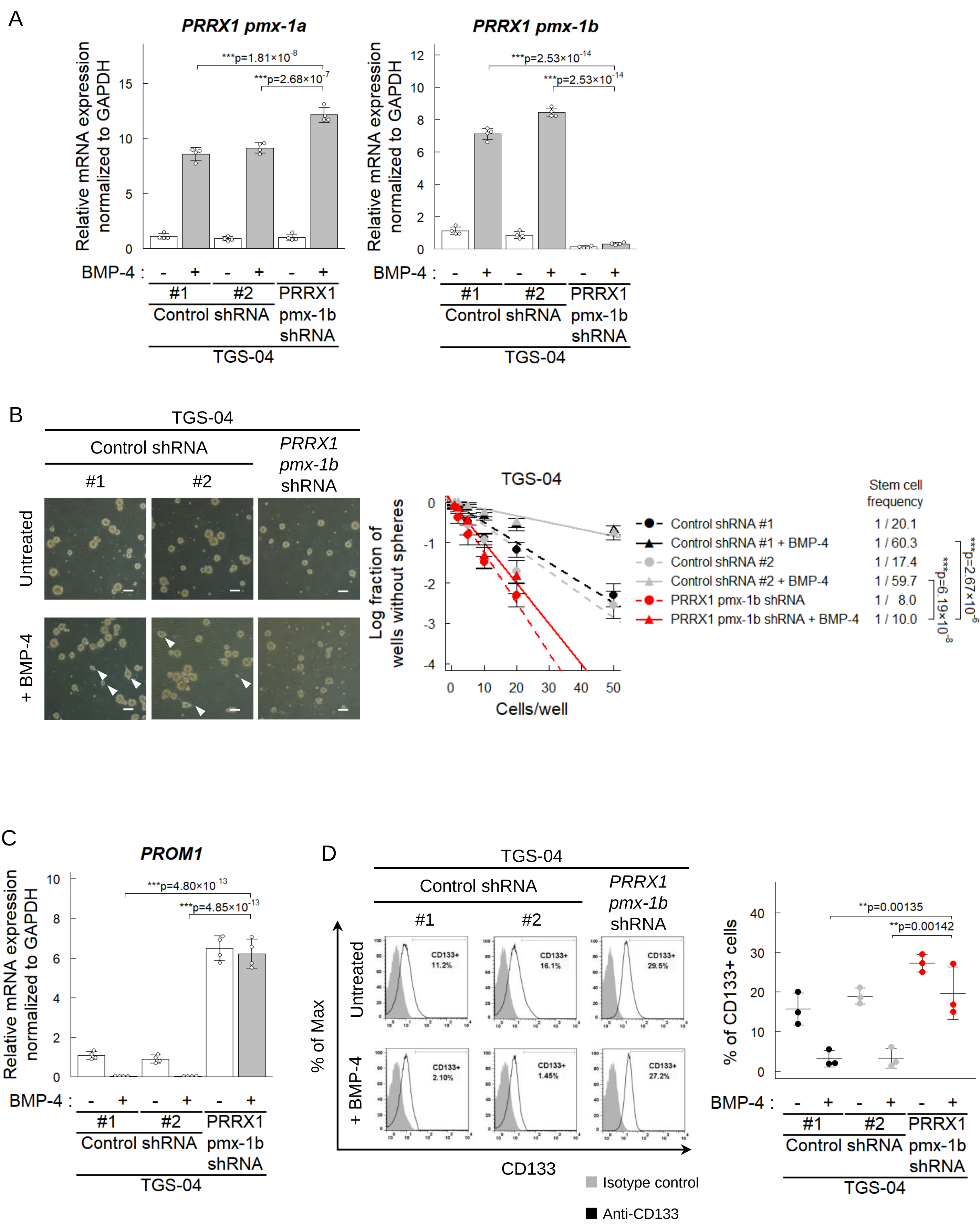

Supplementary Figure S4.

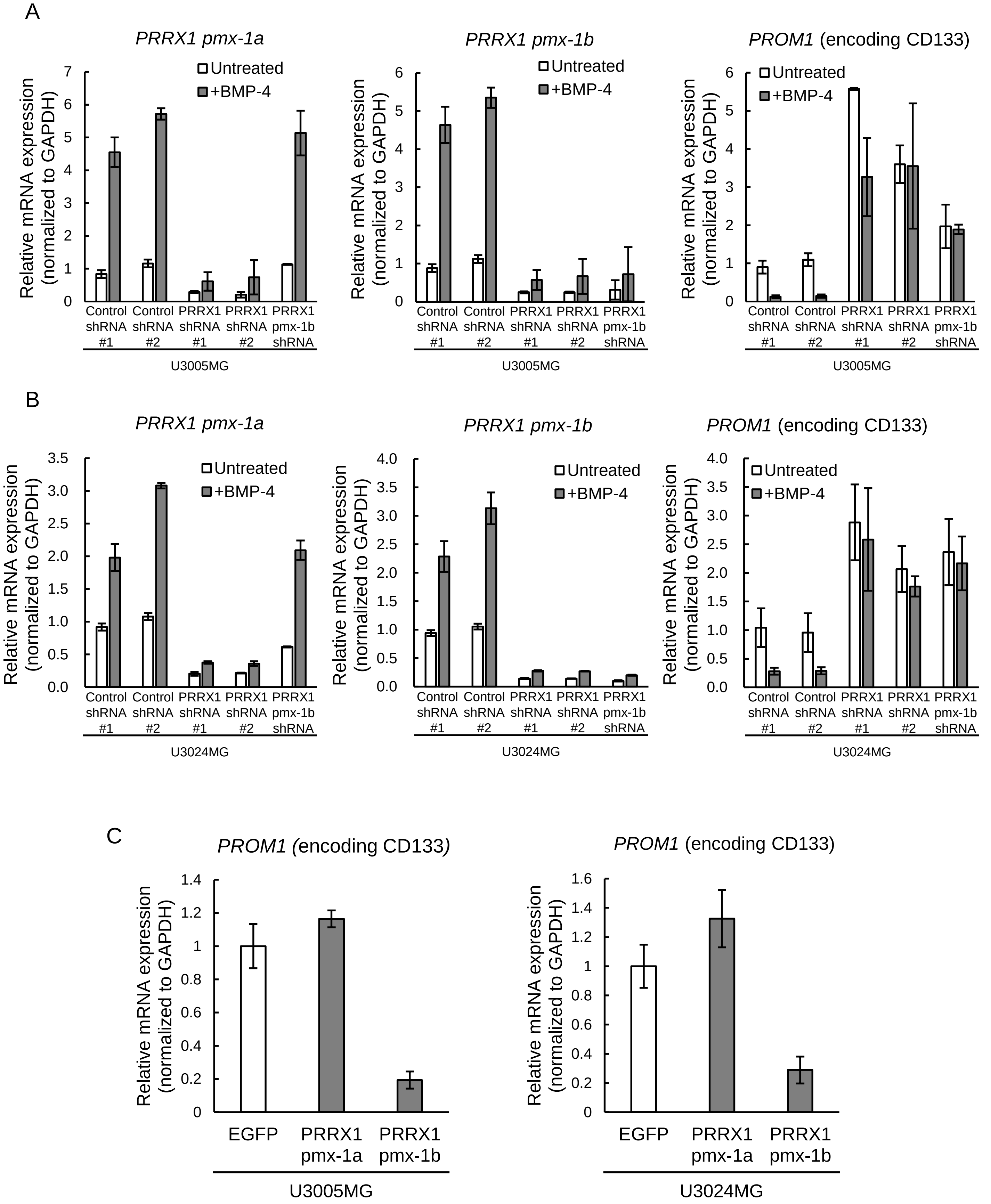

Supplementary Figure S5.

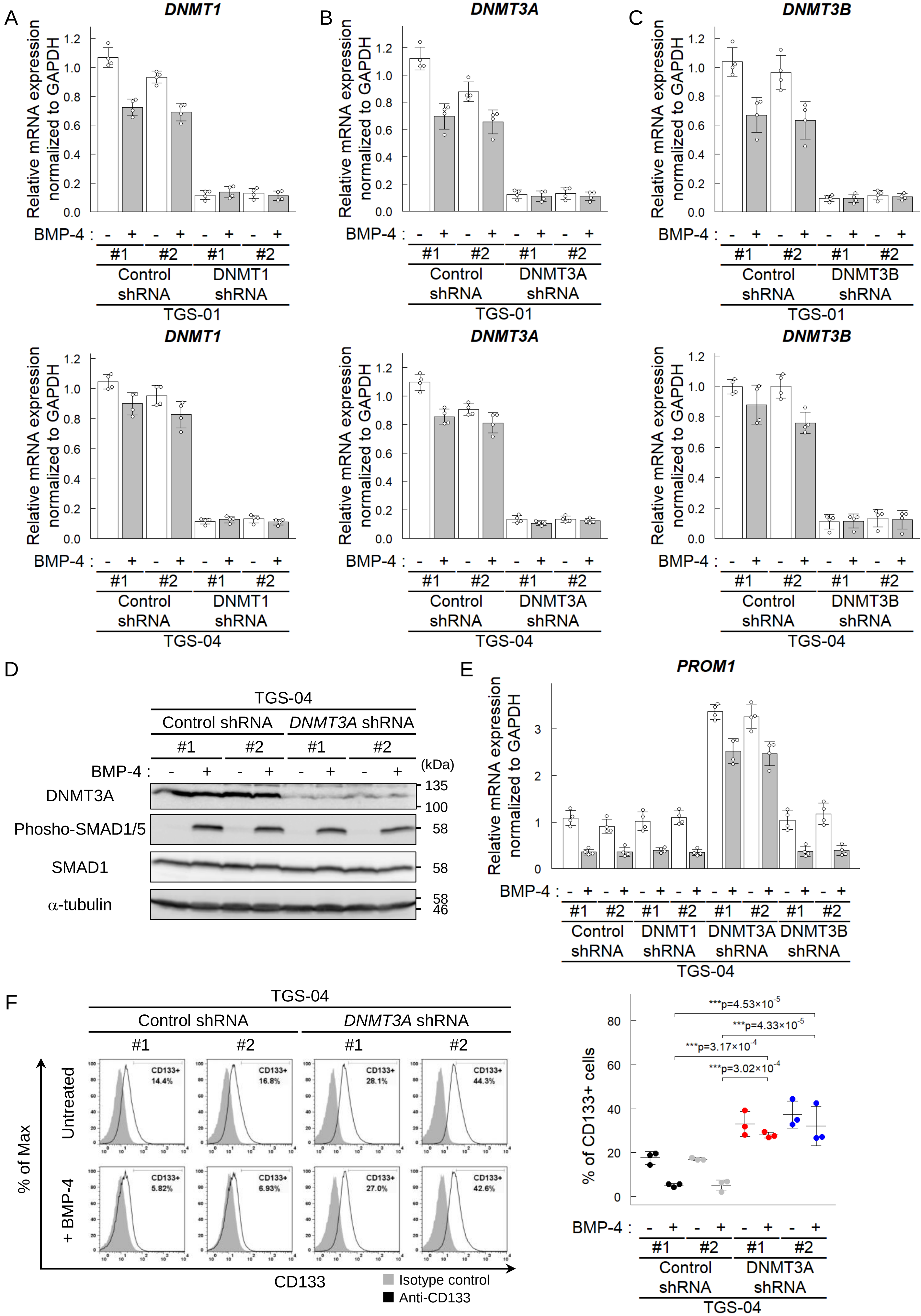

Supplementary Figure S6.

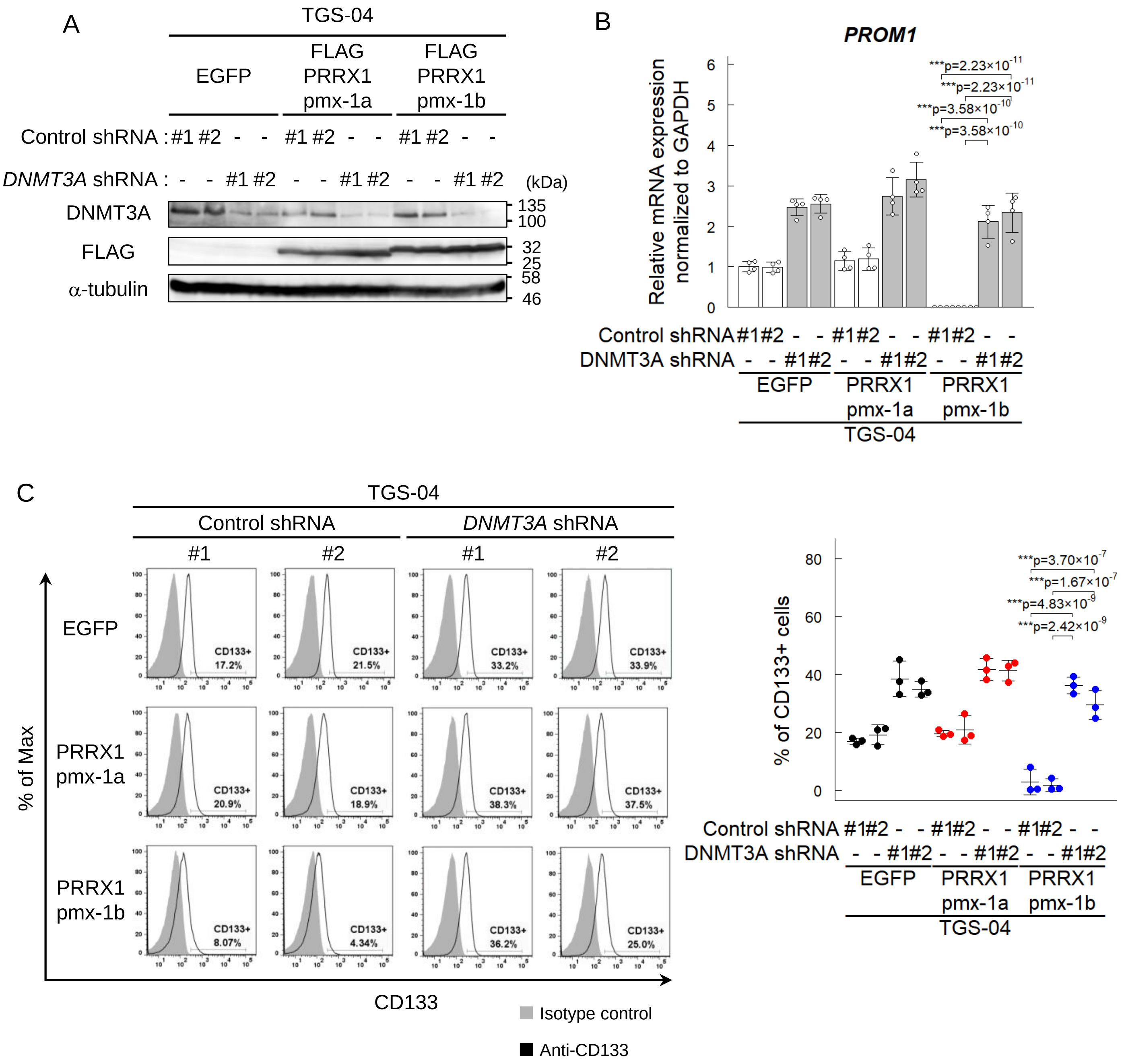

Supplement: Supplementary file 1 — Fig. S1. PRRX1 binds to the PROM1 promoter and negatively modulates its promoter activity. Fig. S2. PRRX1 is required for BMP‐induced loss of the CD133‐positive GIC population. Fig. S3. The PRRX1 pmx‐1b isoform is important for the decrease in the CD133‐positive population of GICs. Fig. S4. Upregulation of PROM1 mRNA by treatment with shRNA for PRRX1 or the pmx‐1b isoform in U3005MG and U3024MG cells. Fig. S5. DNMT3A is involved in the reduction of CD133 expression by BMP signaling. Fig. S6. DNMT3A is required for the PRRX1 pmx‐1b isoform to downregulate CD133 expression. [file MOL2-16-269-s001.pdf]
