# Supplementary material for: PRRX1 induced by BMP signaling decreases tumorigenesis by epigenetically regulating glioma‐initiating cell properties via DNA methyltransferase 3A
Source: Mol Oncol. 2021 Jul 16;16(1):269–88. doi: 10.1002/1878-0261.13051 (PMC8732353; doi:10.1002/1878-0261.13051)
Supplement: Supplementary file 2 — Table S1. Characteristics of glioblastoma cells used in the present study. Table S2. Primer sets for real‐time qPCR. Table S3. Target sequences of shRNA. Table S4. Oligonucleotides used for mutagenesis of the PRRX1‐binding sites. Table S5. Primers used for ChIP‐qPCR. Table S6. Primer sets used for bisulfite sequencing. Table S7. Primer sets used for methylation‐specific PCR. [file MOL2-16-269-s002.docx]

**Supplementary information**

**Fig. S1.** PRRX1 binds to the *PROM1* promoter and negatively modulates its promoter activity. (A) Identification of PRRX1 binding sites in the *PROM1* promoter across species by Contra v2 (<http://bioit.irc.ugent.be/contrav2/index.php>). (B) Relative promoter activity measured in TGS-04 cells treated with or without BMP-4 (30 ng/mL) for 72 hours by dual-luciferase assay. The *PROM1* promoter was subcloned and the AT-rich sequence of PRRX1 binding sites was deleted or converted into a GC-rich sequence. The graphs represent mean ± SD (n = 3 biological replicates). The p-values were determined by Tukey’s test.

**Fig. S2.** PRRX1 is required for BMP-induced loss of the CD133-positive GIC population. TGS-04 cells were treated for 72 hours with or without BMP-4, except for panel (C). (A, B) Knockdown of *PRRX1* mRNA (A) and protein (B) by shRNA for *PRRX1* in TGS-04 cells. (C) Regulation of sphere-forming ability by knockdown of *PRRX1*. Bright-field images showing the morphology of shRNA-expressing TGS-04 cells treated with or without BMP-4 for 5 days under serum-free condition (left). Arrowheads indicate adherent cells. Scale bars: 200 μm. Sphere-forming ability was determined by limiting dilution assay (middle, n = 3 independent experiments) and the sizes of spheres (cultured at the initial density of 50 cells/well) were determined (right, n = 25-48 from 3 independent experiments). Cells were treated with or without BMP-4 (30 ng/mL) for 7 days. (D, E) Regulation of *OLIG2* and *SOX2* mRNA expression by silencing of *PRRX1* (n = 4 biological replicates for TGS-01; n = 5 biological replicates for TGS-04). (F) Surface expression of the CD133 protein in TGS-04 cells evaluated by flow cytometric analysis (n = 3 biological replicates). The graphs in panels (A, C-F) represent mean ± SD of biological replicates. The p-values were determined by Tukey’s test.

**Fig. S3.** The PRRX1 pmx-1b isoform is important for the decrease in the CD133-positive population of GICs. TGS-04 cells were treated for 72 hours with or without BMP-4 (30 ng/mL), except for panel (B). (A) Effect of pmx-1b shRNA on *PRRX1 pmx-1a* and *pmx-1b* expression in TGS-04 cells. (B) Effect of knock-down of *PRRX1 pmx-1b* on sphere-formation. Bright-field images showing the morphology of shRNA-expressing TGS-04 cells treated with or without BMP-4 for 5 days under the serum-free condition (left). Arrowheads indicate the adherent cells. Scale bars: 200 μm. Sphere-forming ability was determined by limiting dilution assay (right). Cells were treated with or without BMP-4 (30 ng/mL) for 7 days. (C) Upregulation of *PROM1* mRNA by treatment with shRNA against the *PRRX1 pmx-1b* isoform in TGS-04 cells. (D) Surface expression of CD133 in TGS-04 cells evaluated by flow cytometric analysis. The graphs in (A-D) represent mean ± SD of biological replicates. The p-values were determined by Tukey’s test.

**Fig. S4.** Upregulation of *PROM1* mRNA by treatment with shRNA for *PRRX1* or the *pmx-1b* isoform in U3005MG and U3024MG cells. (A, B) The cells were treated with or without BMP-4 (30 ng/mL) for 48 hours. Expression of pmx-1a and pmx-1b was also determined in U3005MG (A) and U3024MG (B) cells. The graphs represent mean ± SD of biological duplicates in panels (A) and (B). (C) Downregulation of *PROM1* mRNA by overexpression of pmx-1b in U3005MG and U3024MG cells. The graphs represent mean ± SD of three biological replicates.

**Fig. S5.** DNMT3A is involved in the reduction of CD133 expression by BMP signaling. Cells were treated for 72 hours with or without BMP-4 (30 ng/mL). (A-E) Screening for DNA methyltransferases involved in the expression of *PROM1*. *DNMT1*, *DNMT3A*, and *DNMT3B* were knocked down by shRNAs in TGS-04 cells, and the expression levels of each DNA methyltransferase (A-C) and *PROM1* (E) in TGS-04 cells were quantified by real-time qRT-PCR (n = 4 biological replicates). Immunoblot analysis of downregulation of DNMT3A by shRNA in TGS-04 cells (D). (F) Surface expression of CD133 evaluated by flow cytometric analysis (n = 3 biological replicates). TGS-04 cells expressing control or *DNMT3A* shRNA were treated for 72 hours with or without BMP-4 (30 ng/mL). The graph represents mean ± SD of biological replicates. The p-values were determined by Tukey’s test.

**Fig. S6.** DNMT3A is required for the PRRX1 pmx-1b isoform to downregulate CD133 expression. DNMT3A shRNA and FLAG-tagged PRRX1 were transduced into TGS-04. (A) Immunoblot analysis showing the expression of DNMT3A and FLAG-tagged PRRX1 in TGS-04 cells. (B) Expression of *PROM1* in TGS-04 cells expressing DNMT3A shRNA and PRRX1 (n = 4 biological replicates). (C) Surface expression of CD133 evaluated by flow cytometric analysis in TGS-04 cells (n = 3 biological replicates). The graphs in panels (B, C) represent mean ± SD of biological replicates. The p-values were determined by Tukey’s test.

**Table S1.** Characteristics of glioblastoma cells used in the present study.

| Sample | Age | Sex | Subtype*^1^ | *EGFR* amplification | *TP53* mutation | *IDH1/2* mutation | 1p/19q LOH |
| --- | --- | --- | --- | --- | --- | --- | --- |
| TGS-01 | 54 | M | Mesenchymal | + | - | - | - |
| TGS-04 | 68 | M | Mesenchymal | - | + | - | - |

*^1^According to the classification by Verhaak et al. (2010)

**Table S2.** Primer sets for real-time qPCR

| Target gene | Forward / Reverse | Sequence (5’ → 3’) |
| --- | --- | --- |
| *GAPDH* | Forward | GAAGGTGAAGGTCGGAGTC |
| *GAPDH* | Reverse | GAAGATGGTGATGGGATTTC |
| *PROM1* | Forward | TGGATGCAGAACTTGACAACGT |
| *PROM1* | Reverse | ATACCTGCTACGACAGTCGTGGT |
| *OLIG2* | Forward | CAGAAGCGCTGATGG |
| *OLIG2* | Reverse | TCGGCAGTTTTGGGT |
| *SOX2* | Forward | TGCGAGCGCTGCACAT |
| *SOX2* | Reverse | TCATGAGCGTCTTGGTTTTCC |
| *PRRX1* | Forward | CCAGCTGCAGGCTTTGGA |
| *PRRX1* | Reverse | CGCACAAAAGCATCAGGATAGT |
| *PRRX1* *pmx-1a* | Forward | CATCGTACCTCGTCCTGCTC |
| *PRRX1 pmx-1a* | Reverse | GCCCCTCGTGTAAACAACATC |
| *PRRX1 pmx-1b* | Forward | AATCCTACTCAGGAGACGTGACTG |
| *PRRX1 pmx-1b* | Reverse | AATAAGTAGCCATGGCGCTGTACG |
| *DNMT1* | Forward | CAAACCCCTTTCCAAACCTC |
| *DNMT1* | Reverse | TAATCCTGGGGCTAGGTGAA |
| *DNMT3A* | Forward | ATGTGCGGAAACAACAACTG |
| *DNMT3A* | Reverse | TAGCAGTTCCAGGGGTCTTC |
| *DNMT3B* | Forward | AGAGGGACATCTCACGGTTC |
| *DNMT3B* | Reverse | GGTTGCCCCAGAAGTATCG |

**Table S3**. Target sequences of shRNA

| shRNA | Target sequences of shRNA (5’ → 3’) |
| --- | --- |
| Control shRNA #1 | GUGGUUUACAUGUCGACUAA |
| Control shRNA #2 | AUGGUUUACAUGUUGUGUGA |
| *PRRX1* shRNA #1 | GACAAUGACCAGCUGAACUCA |
| *PRRX1* shRNA #2 | GAGAGCCAUGCUAGCCAAUAA |
| *PRRX1* pmx-1b shRNA | GCGUCUCCGUACAGCGCCAUG |
| *DNMT1* shRNA #1 | GCCGAAUACAUUCUGAUGGAU |
| *DNMT1* shRNA #2 | GCCCAAUGAGACUGACAUCAA |
| *DNMT3A* shRNA #1 | GGCACCAGGGGAAGAUCAUGU |
| *DNMT3A* shRNA #2 | GGACCAUUACUACGAGGUCAA |
| *DNMT3B* shRNA #1 | GCCUCAAGACAAAUUGCUAUA |
| *DNMT3B* shRNA #2 | GCCGGCUCUUCUUCGAAUUUU |

**Table S4.** Oligonucleotides used for mutagenesis of the PRRX1 binding sites

| PRRX1 binding sites | Sequence (5’ → 3’) |
| --- | --- |
| -1032 to -1016 (sense) | CTCTCCCAGTGAAGGCGCCTTCAATATGAGATTTG |
| -1032 to -1016 (antisense) | CAAATCTCATATTGAAGGCGCCTTCACTGGGAGAG |
| -1586 to -1570 (sense) | GCAAAATGAAGACAAGGCGCCCTCACAGGGCTC |
| -1586 to -1570 (antisense) | GAGCCCTGTGAGGGCGCCTTGTCTTCATTTTGC |

**Table S5.** Primers used for ChIP-qPCR

| Location from the *PROM1* transcription start site | Forward /  Reverse | Sequence (5’ → 3’) |
| --- | --- | --- |
| -1603 to -1465 | Forward | TGGGTCCTGATCTCCGTCAA |
| -1603 to -1465 | Reverse | AGGAATGAAAATGAGCCCTG |
| -1083 to -945 | Forward | AGTACAGTAGGACATGGCAA |
| -1083 to -945 | Reverse | AGAACAGCAAGGGAGAAATC |
| -2656 to -2518 (negative control) | Forward | AAACAGTCTGGGCAGGAC |
| -2656 to -2518 (negative control) | Reverse | TATACTGGGTCTCACCTGAA |

**Table S6.** Primer sets used for bisulfite sequencing

| Target | Forward / Reverse | Sequence (5’ → 3’) |
| --- | --- | --- |
| *PROM1* promoter | Forward | TTTATTTTTTGTAGAGGTGTTTG |
| *PROM1* promoter | Reverse | ATTTCCACCACTCAACTAAATA |

**Table S7.** Primer sets used for methylation-specific PCR

| Methylation status | Forward / Reverse | Sequence (5’ → 3’) |
| --- | --- | --- |
| Methylation | Forward | TGGGTCCTGATCTCCGTCAA |
| Methylation | Reverse | AGGAATGAAAATGAGCCCTG |
| Unmethylation | Forward | AAACAGTCTGGGCAGGAC |
| Unmethylation | Reverse | TATACTGGGTCTCACCTGAA |
